# PROTEIN TARGETING TO STARCH Is Required for Localising GRANULE-BOUND STARCH SYNTHASE to Starch Granules and for Normal Amylose Synthesis in Arabidopsis

**DOI:** 10.1371/journal.pbio.1002080

**Published:** 2015-02-24

**Authors:** David Seung, Sebastian Soyk, Mario Coiro, Benjamin A. Maier, Simona Eicke, Samuel C. Zeeman

**Affiliations:** Institute of Agricultural Sciences, ETH Zurich, Zurich, Switzerland; University of Massachusetts at Amherst, UNITED STATES

## Abstract

The domestication of starch crops underpinned the development of human civilisation, yet we still do not fully understand how plants make starch. Starch is composed of glucose polymers that are branched (amylopectin) or linear (amylose). The amount of amylose strongly influences the physico-chemical behaviour of starchy foods during cooking and of starch mixtures in non-food manufacturing processes. The GRANULE-BOUND STARCH SYNTHASE (GBSS) is the glucosyltransferase specifically responsible for elongating amylose polymers and was the only protein known to be required for its biosynthesis. Here, we demonstrate that PROTEIN TARGETING TO STARCH (PTST) is also specifically required for amylose synthesis in Arabidopsis. PTST is a plastidial protein possessing an N-terminal coiled coil domain and a C-terminal carbohydrate binding module (CBM). We discovered that Arabidopsis *ptst* mutants synthesise amylose-free starch and are phenotypically similar to mutants lacking GBSS. Analysis of granule-bound proteins showed a dramatic reduction of GBSS protein in *ptst* mutant starch granules. Pull-down assays with recombinant proteins *in vitro*, as well as immunoprecipitation assays *in planta*, revealed that GBSS physically interacts with PTST via a coiled coil. Furthermore, we show that the CBM domain of PTST, which mediates its interaction with starch granules, is also required for correct GBSS localisation. Fluorescently tagged Arabidopsis GBSS, expressed either in tobacco or Arabidopsis leaves, required the presence of Arabidopsis PTST to localise to starch granules. Mutation of the CBM of PTST caused GBSS to remain in the plastid stroma. PTST fulfils a previously unknown function in targeting GBSS to starch. This sheds new light on the importance of targeting biosynthetic enzymes to sub-cellular sites where their action is required. Importantly, PTST represents a promising new gene target for the biotechnological modification of starch composition, as it is exclusively involved in amylose synthesis.

## Abstract 2

The biosynthesis of starch in plant chloroplasts depends on a novel protein that targets starch synthase to the growing starch granules; this represents a potential target for the biotechnological modification of starch. Read the Synopsis.

## Author Summary

Starch plays a vital role in our everyday lives. It is not only a major dietary carbohydrate, but is also used to manufacture household products such as pharmaceuticals, paper, and textiles. Plants produce starch as a means of storing energy; it is composed of two glucose polymers—amylopectin and amylose. While amylose is present in a smaller quantity than amylopectin, it has a major impact on starch processing. Being able to control the amount of amylose is therefore a goal for biotechnology. Amylose is made by the enzyme GRANULE BOUND STARCH SYNTHASE (GBSS), which was for decades believed to be the only protein required for amylose production. We now report here that a second protein, PROTEIN TARGETING TO STARCH (PTST), is involved in the process. Mutants lacking the PTST protein in the model plant *Arabidopsis thaliana* fail to make any amylose in starch. This is because the GBSS protein, which normally binds to starch, cannot bind in the absence of PTST. This discovery sheds new light on a previously unknown protein targeting process by which enzymes are delivered to the starch. Furthermore, our discovery highlights PTST an ideal target gene for biotechnology.

## Introduction

Starch is a vitally important plant-derived material that is widely used as food and in manufacturing of non-food products. Many plants accumulate starch during photosynthesis within leaf chloroplasts, where it acts as a major storage carbohydrate. The degradation of starch at night provides carbohydrates to fuel respiration and growth when photosynthesis is not possible [1]. Starch is also present in many seeds and storage organs (such as tubers and storage roots) where it serves as an energy reserve to fuel seedling establishment, plant regeneration, or survival under stress conditions.

In its native form, starch exists as semi-crystalline, insoluble granules that are typically between 1 to 100 μm in diameter, depending on botanical source [2]. It is composed of polymers of glucose, in which α-1,4 bonds connect the glucosyl residues into linear chains and α-1,6 bonds form branch points. Two distinct polymers exist within the starch granule—amylose and amylopectin. Amylose is composed of long linear chains with few branch points, while amylopectin has shorter chains and many more branch points. The branches in amylopectin are arranged in a clustered structure that allows adjacent chains to form double helices [3]. The packing of these helices result in the crystalline regions of starch. Amylose is believed to reside in less-crystalline (amorphous) zones inside the starch granule, such as the space between clusters of amylopectin helices. There is significant variation in the amylose content of starch between species, as well as between varieties or cultivars within species. Typically, starch from Arabidopsis leaves contains about 6% amylose [4], while starch in most cereals and storage organs contain about 20%–30% [5].

Detailed information on the biochemistry of starch synthesis can be found in recent reviews [3,6]. Briefly, the synthesis of amylopectin requires the co-ordinated activities of four soluble starch synthase classes (SS1, SS2, SS3, SS4) that initiate glucan chains and elongate them using the glucose donor molecule, ADP-Glucose (ADP-Glc). Branching enzyme activity is required to introduce α-1,6 branch points, while specialised debranching enzymes are thought to subsequently remove misplaced branch points to promote crystallisation. Amylopectin clusters are radially oriented within the starch granule, and its synthesis occurs at the granule surface. In contrast, amylose is synthesised from within the amylopectin matrix [7,8]. This is achieved by the activity of the GRANULE-BOUND STARCH SYNTHASE (GBSS), the only starch synthase isoform required for amylose synthesis. Mutants carrying defects in the GBSS (or “waxy”) gene have been isolated in many species, including Arabidopsis [9,10], maize [11], rice [12], cassava [13], and potato [14], all of which produce amylose-free starch granules. Several properties distinguish GBSS from other starch synthase isoforms. Firstly, it is tightly bound to starch granules and is the most abundant protein encapsulated within starch [15]. Secondly, unbound GBSS protein appears to be unstable, since it is not detectable in soluble protein fractions of leaves, even in the absence of starch granules (e.g., at the end of the night, when the starch has been fully degraded) [16]. Finally, unlike soluble starch synthase isoforms such as SS2, GBSS elongates glucans processively—adding more than one glucosyl monomer per substrate encounter [17]. These properties in combination allow GBSS within starch granules to elongate long amylose chains that are presumably not accessible to soluble branching enzymes in the stroma.

Amylose content has a major influence on the physico-chemical behaviour of starch and is one of the most important parameters determining starch suitability for different applications [18,19]. During cooking or industrial processing, amylose content influences both gel firmness and stickiness. Starches with no amylose, such as waxy corn starch, are extensively used by the food industry to achieve desirable food textures [20]. Furthermore, waxy starches are used in paper manufacturing, where clear, consistent gels are required. Conversely, high amylose starches have also received market interest as its consumption may provide health benefits. High amylose starch is less-readily digested in the gut, and may lower the glycemic index of foods, while possibly contributing to dietary fiber intake [21]. The commercial interest in starches with modified amylose content has driven the application of biotechnological tools to alter amylose content *in planta* [21–25]. However, the GBSS gene has so far been the only target for direct control of amylose content without affecting amylopectin.

Here, we reveal a previously unknown process for targeting proteins to starch, and show that it is essential for normal rates of amylose synthesis in Arabidopsis. We investigated the function of a previously identified chloroplastic “scaffold protein” encoded by locus At5g39790 and proposed to be involved in starch metabolism [26]. This protein has starch-binding activity and possesses a family 48 carbohydrate binding module (CBM48) towards its C-terminus [26]. Other enzymes involved in starch metabolism, such as branching and debranching enzymes [27], and the glucan phosphatase SEX4 [28], also posess family 48 CBMs. While the previous study did not identify the function of the At5g39790 protein, they speculated that it could play a scaffolding role, anchoring other proteins to starch, since in addition to its CBM48, it has a predicted coiled coil motif; a common structural feature associated with protein-protein interactions [29]. Using co-expression and protein abundance analysis, the study identified a number of starch metabolic enzymes as potential interaction partners for the At5g39790 protein [26]. These included GBSS, which the authors speculated may be anchored to the starch granule via the scaffold protein.

In this study, we used molecular genetic and biochemical approaches to demonstrate that the protein encoded by At5g39790 is involved in targeting GBSS to the starch granule, and thus plays a crucial role in amylose synthesis. We hence designate it PROTEIN TARGETING TO STARCH (PTST). GBSS fails to localise to the starch granule in Arabidopsis mutants lacking PTST, and the mutants synthesise amylose-free starch. We show that PTST interacts directly with both GBSS and the starch granule. However, the majority of PTST protein is present in the stroma as a soluble protein, and does not stably anchor GBSS to starch. We propose that PTST mediates a dynamic protein transport/delivery mechanism, and interacts with starch transiently rather than acting as a scaffold. We discuss the significance of these findings in terms of the new insight into intracellular protein targeting, and in terms of the potential biotechnological modification of starch.

## Results

### PTST Is a Highly Conserved Plant Protein

**Fig 1:**
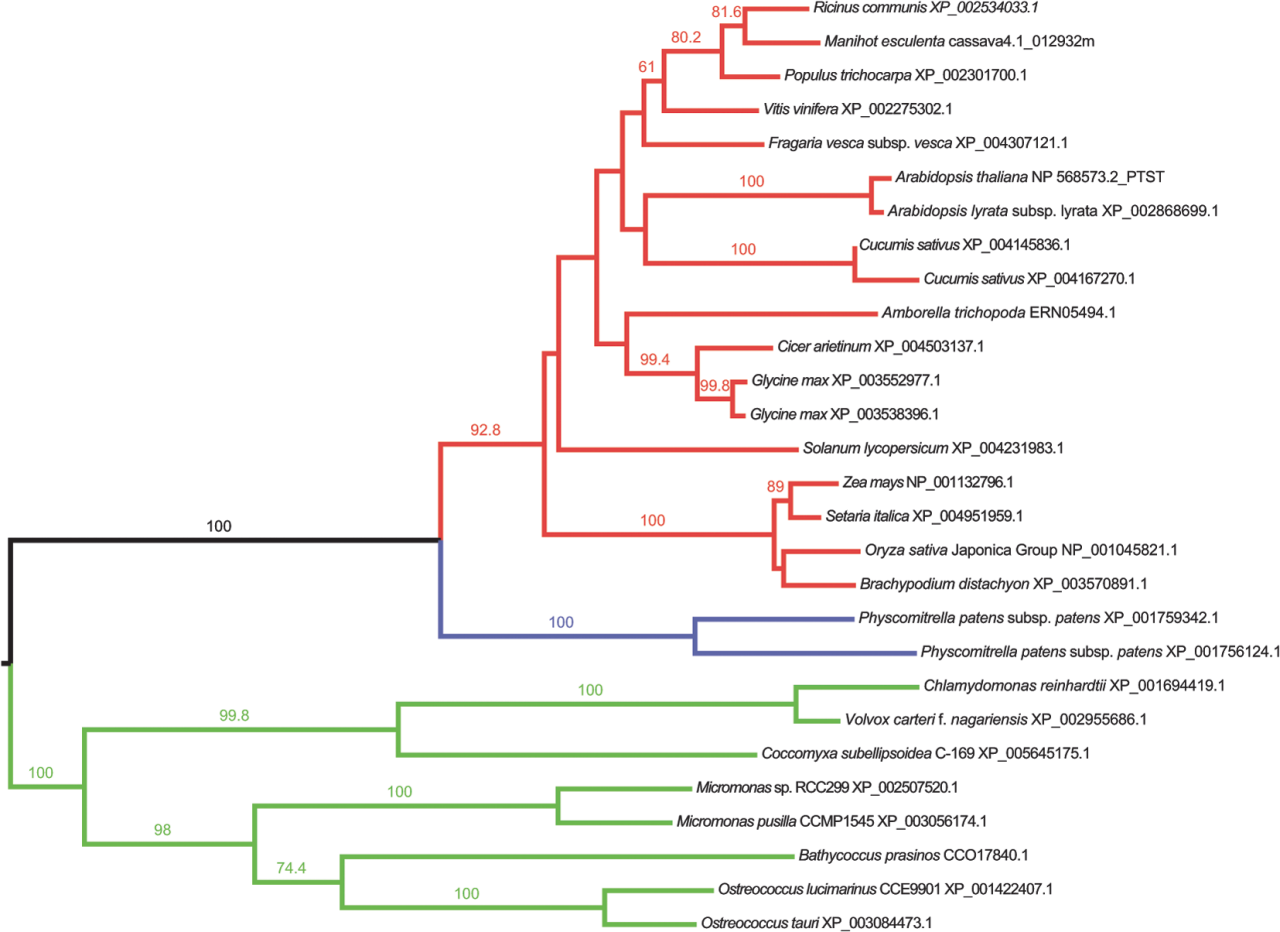
Maximum likelihood phylogenetic tree of PTST proteins from the MAFFT alignment. Angiosperm sequences are shown in red, mosses in blue, while chlorophyte sequences are shown in green. Bootstrap values that are >50 are shown over the branches. The alignment used to generate this tree is available as S1 Data.

The PTST protein was previously identified as a potential candidate for involvement in starch metabolism [26]. First, we performed a phylogenetic analysis on PTST protein sequences to assess its conservation among plant species (Fig. 1). PTST was found as a single copy gene in Arabidopsis encoded by locus At5g39790. PTST sequences were highly conserved, and orthologs were found in both Embryophytes (land plants) and the Chlorophyte branch of the green algae. This suggests that the protein originated with the green lineage. Furthermore, there was a high degree of conservation among angiosperm sequences, suggesting that PTST may play an important role in this group.

### Knockout Mutations of PTST in Arabidopsis Result in the Production of Amylose-Free Starch

**Fig 2:**
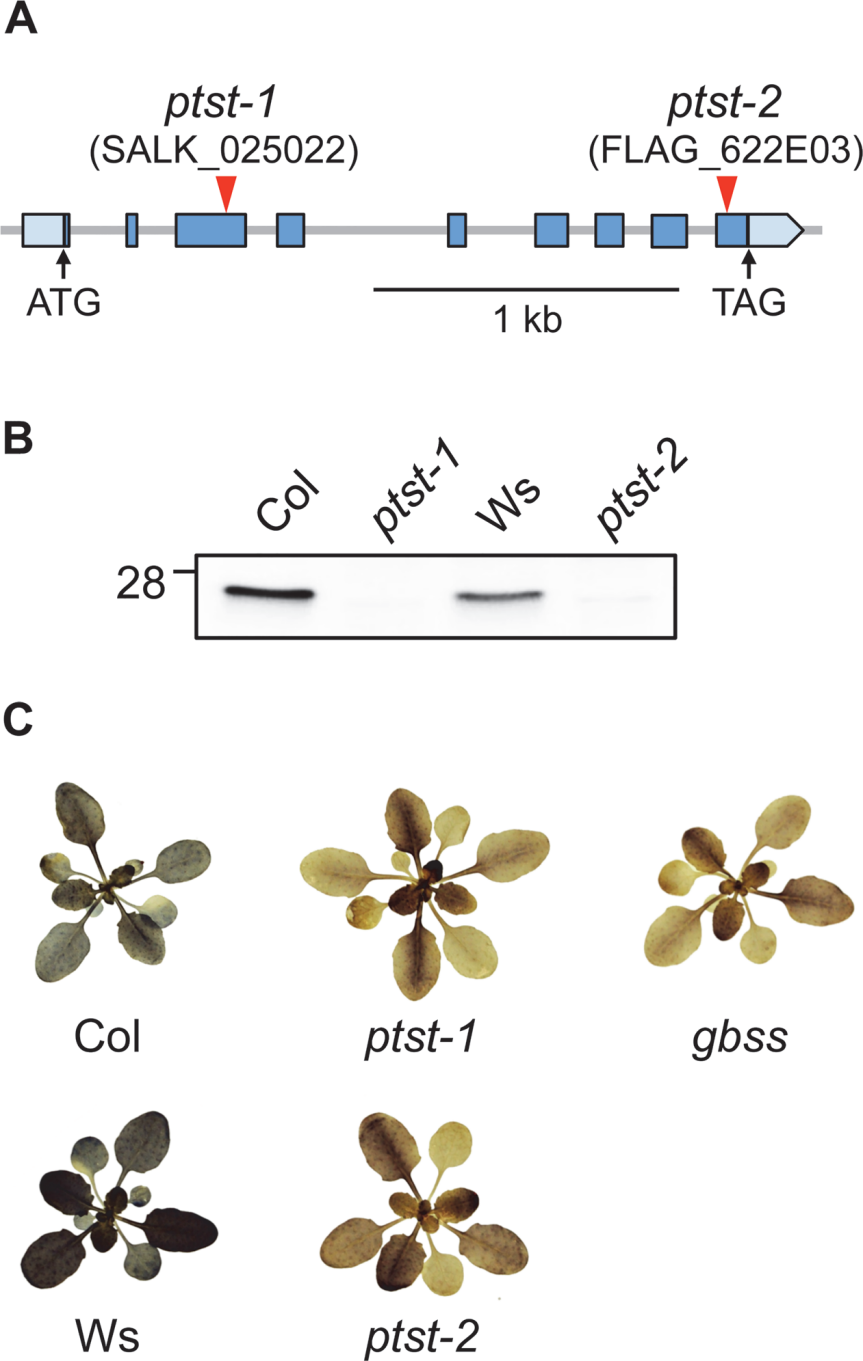
*ptst* knockout mutants produce amylose-free starch. (A) Schematic illustration of the exon-intron structure of the *PTST* gene. Exons are represented by blue boxes. Pale blue boxes represent the 5′ and 3′ UTRs. Translation start (ATG) and stop (TAG) codons are indicated with arrows. Red arrows indicate T-DNA insertion sites. (B) Immunoblot detection of PTST in soluble protein extracts from leaves. The corresponding wild types to *ptst-1* and *ptst-2* are Columbia (Col) and Wassilewskija (Ws), respectively. (C) Representative images of 4-week-old rosettes. Plants harvested at the end of the photoperiod were cleared of chlorophyll and iodine-stained to visualise starch. Amylose-free mutants (*gbss*, *ptst-1*, and *ptst-2*) produce a brown staining that is distinct from the wild types.

To investigate the role of PTST in starch metabolism, we obtained Arabidopsis mutant lines carrying T-DNA insertions in the PTST gene. We isolated two independent homozygous knockout lines, designated *ptst-1* and *ptst-2*, carrying T-DNA insertions in exons 3 and 9, respectively (Fig. 2A). The exact insertion sites are presented in S1 Table. The *ptst*-*1* mutant is in the Columbia (“Col”) ecotype background while *ptst-2* is in the Wassilewskija (“Ws”) background. We analysed soluble protein extracts from leaves by immunoblotting, using antibodies raised against the recombinant Arabidopsis PTST protein. Bands corresponding to PTST at the expected molecular weight of 26 kDa were detected in wild-type Col and Ws extracts, but not in the *ptst* mutants (Fig. 2B), confirming that both alleles are knockouts.

To investigate whether the *ptst* mutations have an impact on starch biosynthesis, plants were grown under a 12-h/12-h day/night cycle. Entire rosettes were harvested at the end of the day and stained for starch using an iodine/potassium iodide solution. Both Col and Ws wild-type plants stained a distinctive blue-brown colour, indicating the presence of amylose-containing starch. However, plants from both *ptst* knockout lines stained only brown, suggesting the absence of amylose. Indeed the staining was very similar to the amylose-free *gbss* mutant (Fig. 2C). The total starch content in leaves at this time was unaltered in the *ptst* and *gbss* mutants compared to the respective wild types (Table 1). Furthermore, starch content analysis over a 24 h period showed that the total amount of starch was not altered in either the *ptst-1* or the *gbss* mutant at any time point (S1 Fig.). These data show that the altered staining of *ptst* does not simply reflect a change in the amount of starch. We further quantified the amylose content of purified starch granules using an iodine colourimetry-based method [30]. The percentage of amylose in starch was approximately 8% and 11% for wild-type Col and Ws starch, respectively (Table 1). However, both *ptst* mutant alleles had an amylose content that was below the detection limit of our assay. Additionally, gel permeation chromatography of dispersed starch granules over a Sepharose CL-2B column confirmed that the amylose fraction was missing from *ptst* starch granules (S2A Fig.). These results demonstrate that the loss of PTST results in the production of amylose-free starch.

**Table 1:** Total starch and amylose content of *ptst* mutants.

Ecotype	Genotype	Total Starch Content:		Apparent Amylose Content:
		ED	EN	Percent of Total Starch
Col-0	WT	7.0 ± 0.2	0.2 ± 0.0	7.8 ± 0.1
	*ptst-1*	7.3 ± 0.3	0.1 ± 0.0	Below detection limit
	*gbss*	7.3 ± 0.5	0.1 ± 0.0	Below detection limit
Ws	WT	7.8 ± 0.4	0.2 ± 0.0	11.4 ± 0.1
	*ptst-2*	7.1 ± 0.3	0.1 ± 0.0	Below detection limit

Values for starch content at end of day (ED) and end of night (EN) time points are the mean ± standard error of the mean (SEM) from *n* = 4–5 individual plants. Amylose was measured in isolated starch granules using iodine colourimetric spectra, and represent mean ± SEM from three determinations.

### Loss of PTST Results in a Dramatic Reduction of Starch-Bound GBSS Protein

**Fig 3:**
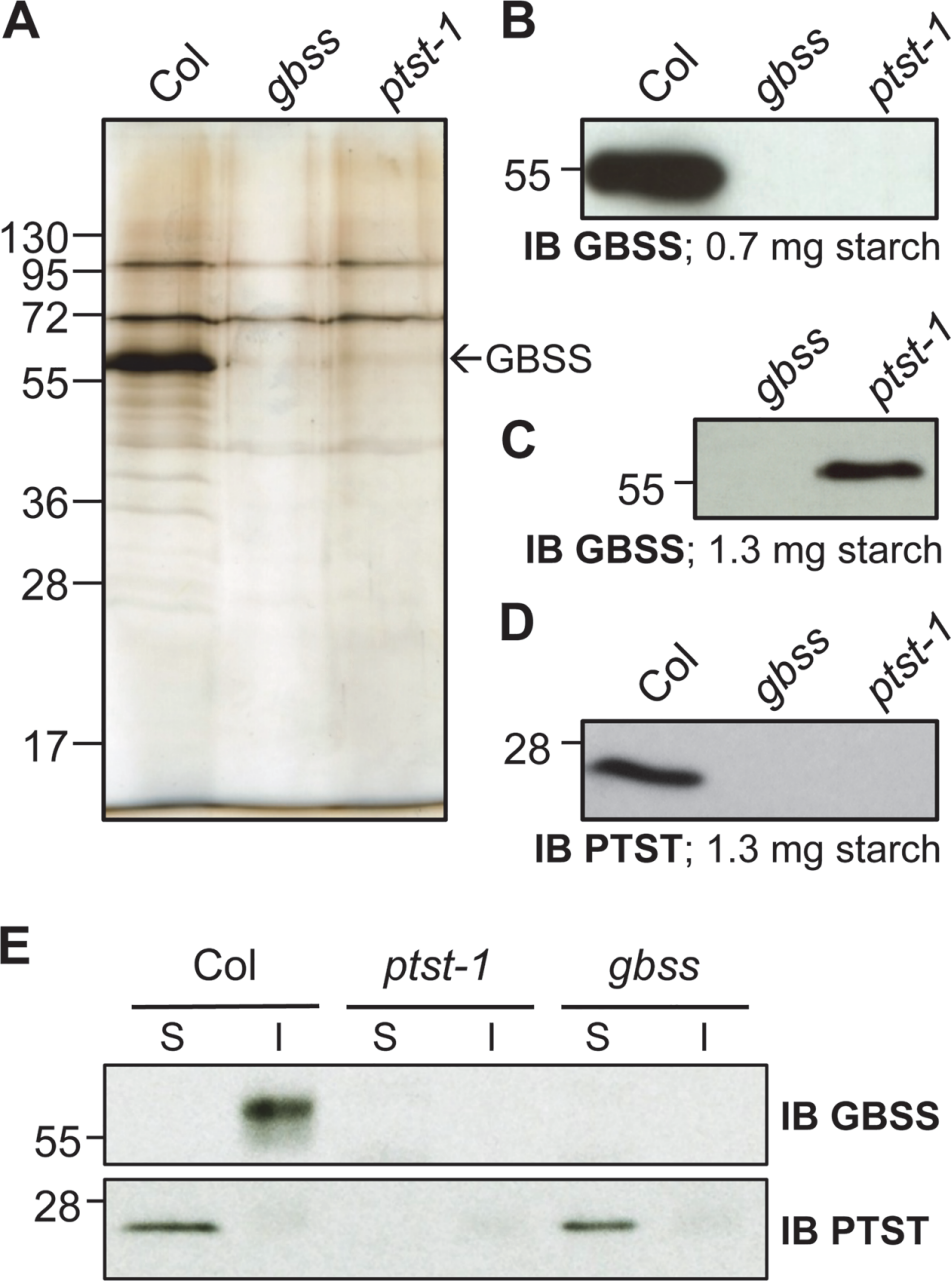
The abundance of granule-bound GBSS protein is greatly reduced in *ptst*. (A) Silver-stained SDS-PAGE gels of granule-bound proteins extracted from purified starch granules. Lanes were loaded according to equivalent mass of starch (1.3 mg starch). The band corresponding to GBSS is indicated. (B) Immunoblot detection of GBSS in granule-bound protein extracts. Loading was according to equivalent mass of starch (0.7 mg). (C) Same as (B), but more extract was loaded (equivalent to1.3 mg starch) and exposure time was greatly extended. (D) Immunoblot detection of PTST in granule-bound protein extracts. (E) Soluble (S) and insoluble (I) protein fractions of leaves were subject to immunoblot analysis with GBSS and PTST antibodies. Starch-bound proteins are contained in the insoluble fraction.

GBSS is the key enzyme responsible for amylose synthesis. We investigated whether the abundance of GBSS in starch granules was altered in the *ptst* mutant. Granule-bound proteins were extracted by gelatinising purified starch granules in an SDS-containing medium, and subsequently visualised on silver-stained SDS-PAGE gels. GBSS was the major granule-bound protein, appearing as the dominant band at its expected molecular weight of 59 kDa (Fig. 3A). As expected, this band was absent from the starch of the *gbss* mutant. Surprisingly, the band was also undetectable in the *ptst* mutant. We conducted more sensitive analyses by immunoblotting these granule-bound protein extracts with an antiserum raised against the pea GBSS. A band corresponding to GBSS was detected on *ptst* starch granules, but only under high protein loading and extended exposure times for the blot (Fig. 3B and 3C). This suggests that GBSS is dramatically reduced in abundance. We repeated the immunoblotting experiment with a dilution series of the wild-type granule-bound proteins, comparing it with those from *ptst*, which established that the degree of reduction of GBSS on *ptst* starch granules is about 100-fold (S3 Fig.)

Immunoblotting with a PTST-specific antiserum revealed that some PTST protein was bound to starch granules from wild type, even though there was no obvious band at that location on the silver-stained gel. As expected, this band was absent from the immunoblot of *ptst* starch-bound proteins, but also from those of the *gbss* mutants (Fig. 3D). Hence, the loss of either protein results in the loss of the other protein from starch. This suggests a functional interaction between GBSS and PTST on the granule.

To further investigate the granule-bound localisation of both GBSS and PTST, we sequentially extracted soluble and insoluble proteins from leaves. Granule-bound proteins are present in the insoluble fraction (since starch is insoluble), while free stromal proteins are present in the soluble fraction. The vast majority of PTST protein was in the soluble protein fraction and not granule-bound (Fig. 3E). Additionally, while the abundance of starch-bound PTST was reduced in *gbss* mutants (Fig. 3D), its abundance in the soluble fraction was unaltered (Fig. 3E). In contrast, GBSS was exclusively bound to starch granules in the wild type. Despite the near absence of GBSS from the starch granules of *ptst* mutants, we could not detect it in the soluble fraction, supporting existing speculation that unbound GBSS is degraded [16]. Additionally, we performed qPCR analyses to quantify GBSS transcript levels in *ptst* plants. No differences were observed in the quantity of GBSS transcripts between *ptst* and wild-type plants 2 h into the light period (S2 Data). This is the time point at which circadian clock-regulated GBSS expression is at its maximum level [16]. This strongly suggests that the reduction in GBSS in *ptst* occurs at the post-transcriptional level.

### PTST Affects the Efficiency of GBSS Targeting to the Starch Granule

To further characterise the amylose-free phenotype of *ptst*, we crossed *ptst-1* with two different Arabidopsis mutants that have higher amylose content—*sex4* and *dpe1* [4,31]. SEX4 is a glucan phosphatase required for efficient starch degradation. The *sex4* mutants do not degrade as much starch at night as they make during the day, resulting in the accumulation of starch over multiple diurnal cycles [32]. Amylose can therefore be synthesised over multiple photoperiods in *sex4* starch granules and reach levels exceeding 30% of total granule mass (Table 2), as opposed to wild-type granules where amylose is only synthesised over a single photoperiod and is less than 10% (Tables 1 and 2) [4]. DPE1 is a glucanotransferase involved in plastidial malto-oligosaccaharide metabolism during starch degradation. While *dpe1* mutants also have a mild impairment in the rate of starch degradation, the primary reason for its high amylose content (over 20% of granule mass; see Table 2) is the accumulation of short malto-oligosaccharides, which can act as primers for GBSS activity [31,33].

**Table 2:** Amylose content of *ptst* double mutants.

Experiment	Genotype	Apparent Amylose Content:
		Percent of Total Starch
1	WT	9.7 ± 0.4
	*sex4*	31.5 ± 0.7
	*ptst sex4*	9.1 ± 0.3
	*gbss sex4*	Below detection limit
2	WT	9.0 ± 0.2
	*dpe1*	21.7 ± 0.1
	*dpe1 ptst*	Below detection limit
	*dpe1 gbss*	Below detection limit

Amylose was measured in isolated starch granules using iodine colourimetric spectra and represent mean ± standard error of the mean (SEM) from three determinations.

**Fig 4:**
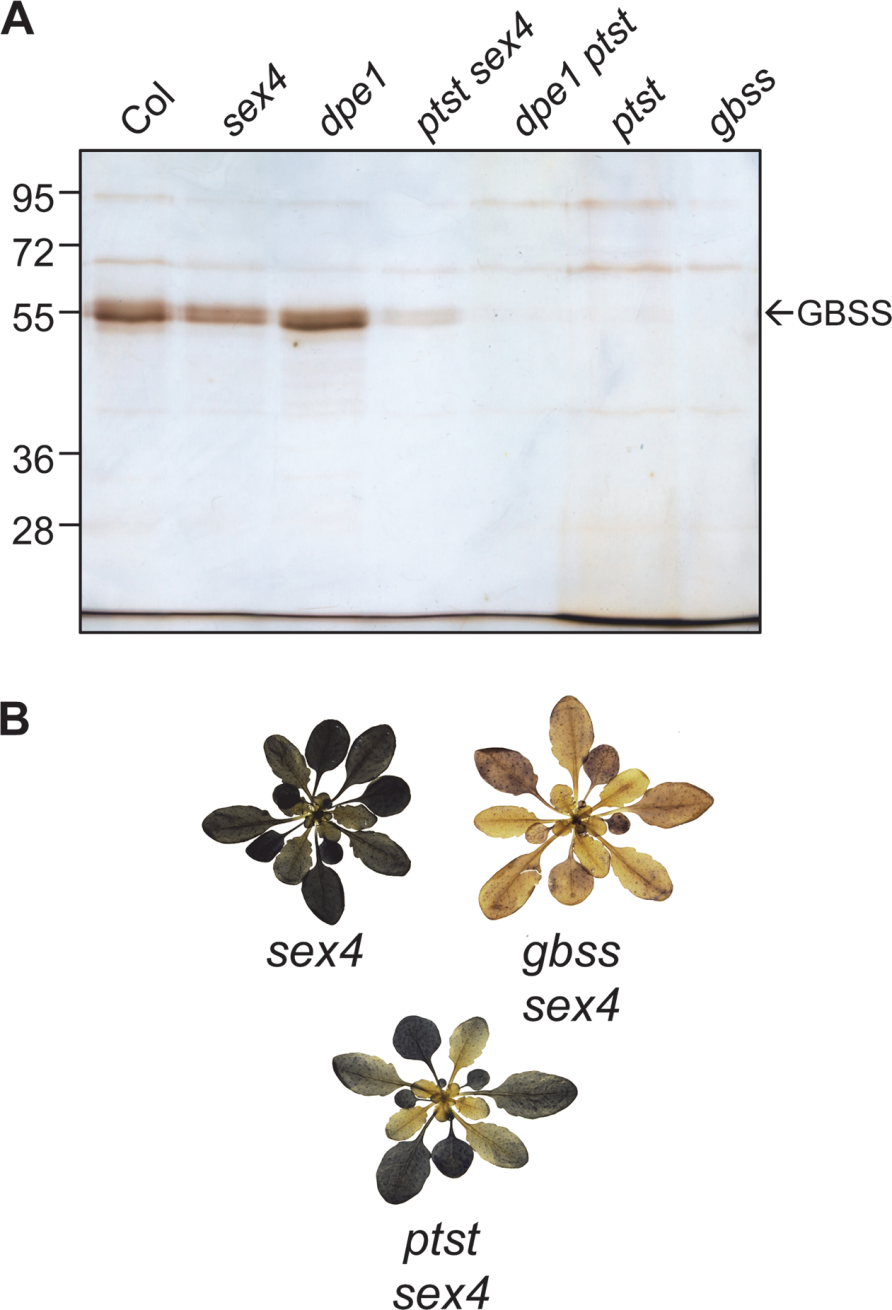
Introduction of the *ptst* allele into high amylose mutants. (A) Silver-stained SDS-PAGE gels of granule-bound proteins from *ptst sex4* and *dpe1 ptst* double mutants. Lanes were loaded according to equivalent mass of starch (1.3 mg starch). (B) Iodine stained rosettes of *ptst sex4* mutants. Note that the younger leaves stain like the amylose-free *gbss sex4* mutant, while older leaves stain darker.

No amylose was detected in the *dpe1 ptst* double mutant, even though *dpe1* single mutants had more than twice the amylose content of the wild type (Table 2). However, the *ptst sex4* double mutant had intermediate levels: the amylose content was only one-third of that found in the *sex4* single mutant but was comparable to the wild type (Table 2). These results were confirmed using Sepharose CL-2B chromatography (S2B Fig.). Silver-stained SDS-PAGE gels of granule-bound proteins revealed that the *dpe1* and *sex4* mutants had GBSS levels similar to the wild type (Fig. 4A). However, no GBSS could be detected on *dpe1 ptst* granules, as for the *ptst* single mutant. In *ptst sex4* granules, GBSS was detectable but still greatly reduced compared with the wild type. These data are consistent with the idea that loss of PTST causes a dramatic reduction in GBSS targeting to starch, and thereby affects the rate of amylose synthesis. The fact that *ptst sex4* had wild-type amylose levels despite having much less GBSS is consistent with the idea that amylose synthesis can proceed over many days, since the loss of SEX4 means that the starch is never fully degraded. Further evidence for this was seen when *ptst sex4* rosettes were stained with iodine (Fig. 4B). Older leaves, where starch has been accumulating for the longest period of time, stained blue due to the presence of amylose, while younger leaves stained brown, suggesting they are amylose-free.

### GBSS and PTST Interact Via Coiled Coils

**Fig 5:**
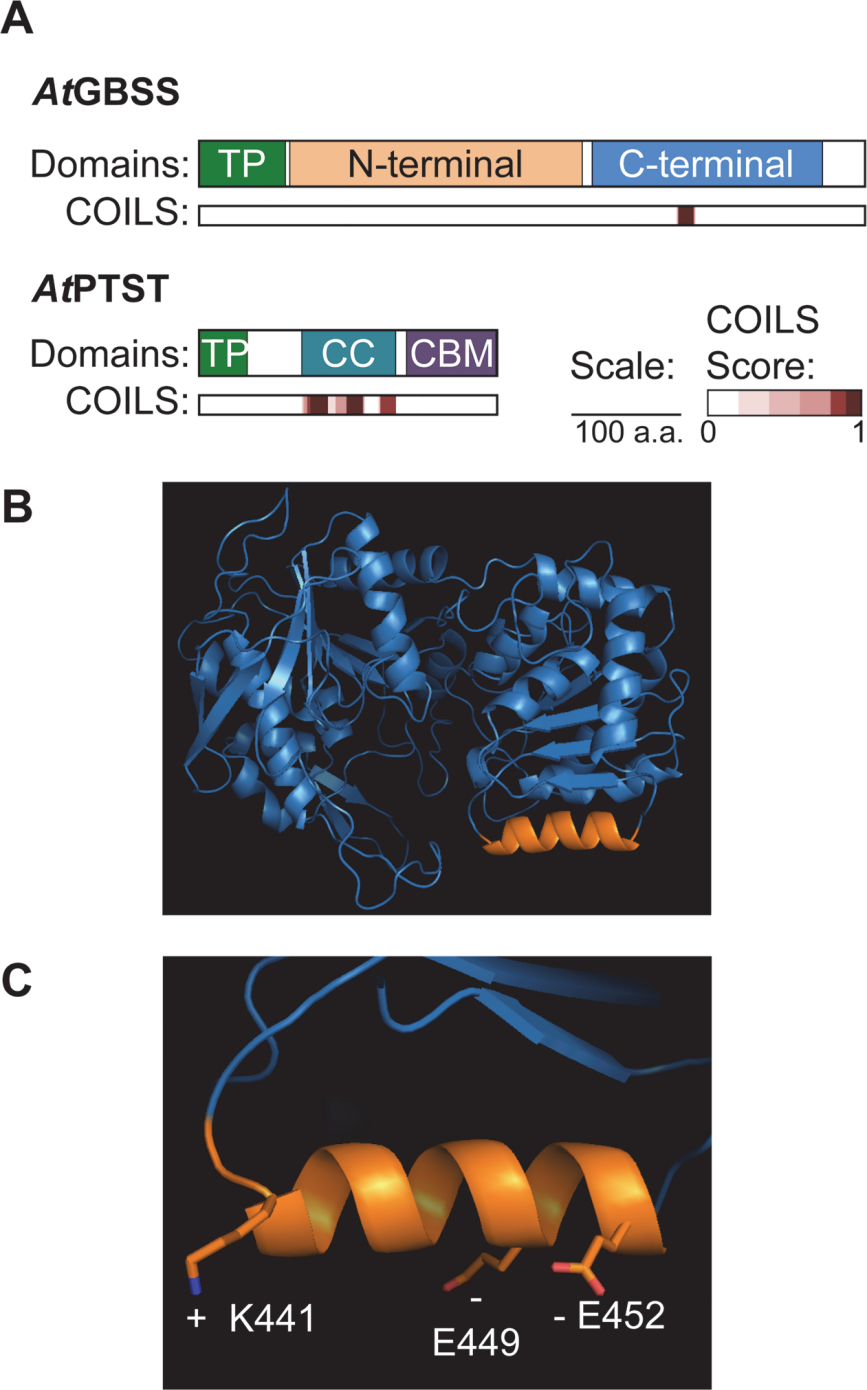
Analysis of PTST and GBSS using bioinformatic tools. (A) COILS analysis to predict coiled coils in Arabidopsis GBSS and PTST sequences. The location of predicted coiled coils are shown with an indication of the probability score (ranging from 0 to 1, where 1 is the highest probability). (B) Homology model of the Arabidopsis GBSS based on the rice GBSS1 crystal structure. The location of the predicted coiled coil from (A) is depicted on the structure in orange. (C) Presence of surface-exposed charged side chains on the coiled coil containing helix.

The PTST protein has two recognisable domains—the coiled coil-containing domain and the CBM48 domain [26]. GBSS is also predicted to possess a coiled coil [26]. Based on the amylose-free phenotype of *ptst* mutants, we hypothesised that GBSS may interact directly with PTST via the coiled coils. The COILS/PCOILS server [34] predicted one coiled coil region on the Arabidopsis GBSS protein—a 14-amino-acid stretch in the C-terminal domain—with a very high probability score (Fig. 5A). This prediction for a coiled coil was conserved in all vascular plant GBSS protein sequences examined (S4A Fig.). Meanwhile, the N-terminal part of PTST was predicted to form three sequential coiled coils.

Using the solved protein structure of rice GBSS as a template [35], we modelled the structure of Arabidopsis GBSS using the SWISS-MODEL automated homology modelling server [36]. We located the predicted coiled coil on the model, and found that it occupied a 13-amino-acid helix exposed on the surface of the C-terminal domain (Fig. 5B). Coiled coil-mediated protein-protein binding typically involve electrostatic interactions between charged amino acids on the coiled coil [29]. While there were numerous charged amino acids present on this helix, some had buried or partially buried side chains (Glu444, Glu451, Lys453) and were unlikely to be available for protein-protein interaction. The side chains of Lys440 and Lys442 were surface exposed, but were most likely occupied in ionic interactions with Glu412 on the adjacent helix. However, three of the amino acids had surface-exposed charges that were not in the proximity of any other charged amino acids for intramolecular ionic interactions. These were Lys441, Glu449, and Glu452 (Fig. 5C). The presence of surface-exposed charges on this helix gives it strong potential for mediating protein-protein interaction.

We investigated whether PTST and GBSS proteins could interact with each other *in vitro*. Furthermore, we sought to determine whether the predicted coiled coil on GBSS participated in such a protein-protein interaction. To address this, we used an *in vitro* pull-down assay using recombinant proteins expressed in *Escherichia coli*. Briefly, GBSS protein with a C-terminal polyhistidine (His_6_) tag was incubated with an N-terminal glutathione-S-transferase (GST)-tagged PTST protein. GBSS was recovered using a Ni^2+^-NTA resin, which specifically binds the His_6_ tag. The GBSS variants tested in this assay included charge-shift mutations of the three surface-exposed charges in the coiled coil-containing helix (Lys441→Glu, Glu449→Lys, and Glu452→Lys). Additionally, to abolish the coiled coil completely, we substituted the coiled coil-containing helix with the homologous helix from Arabidopsis SSI (with no predicted coiled coil), creating a chimeric GBSS protein (see S4B Fig. for more details).

**Fig 6:**
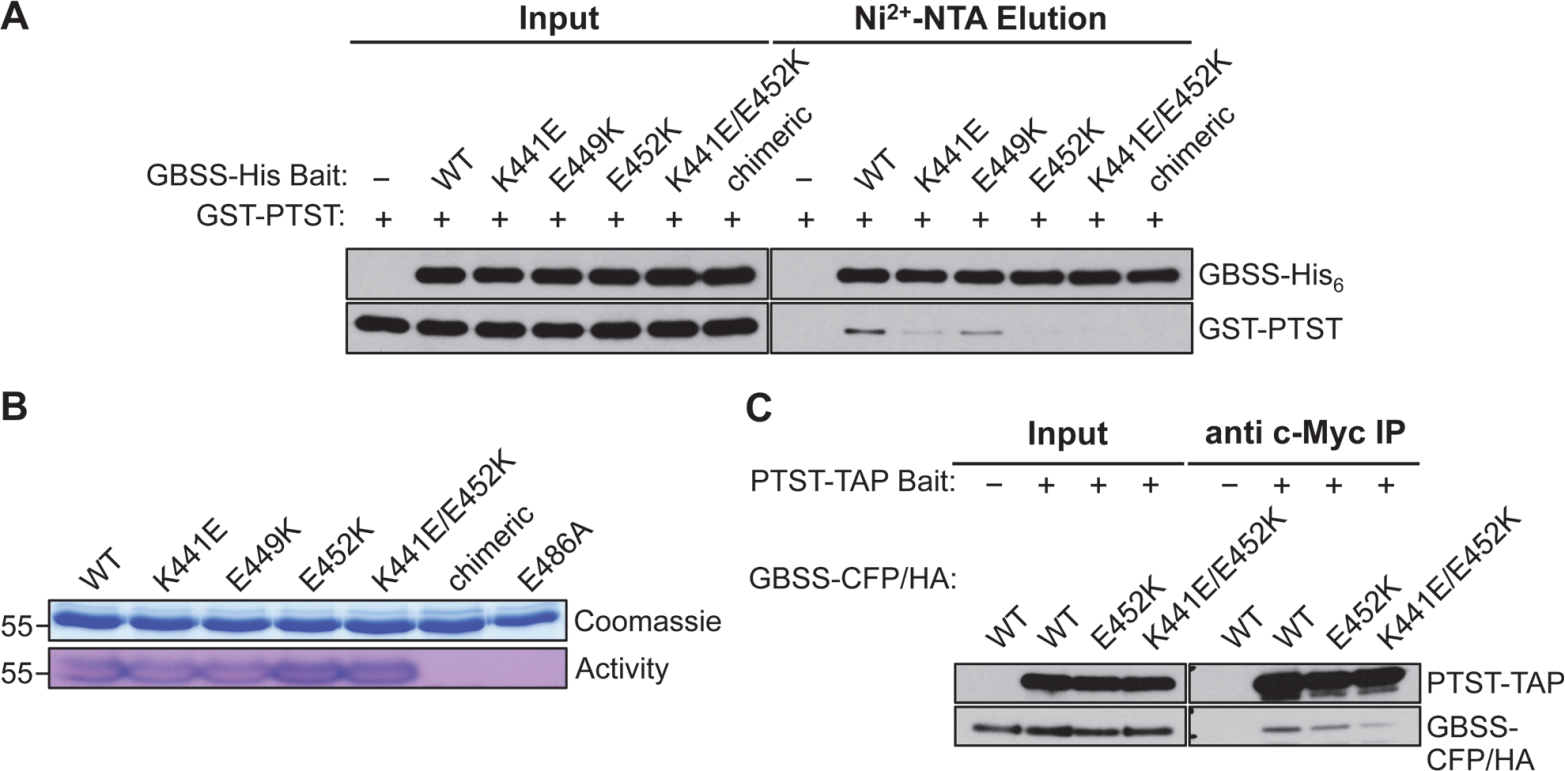
GBSS interacts with PTST through its coiled coil. (A) *In vitro* pulldown assay using Ni^2+^-NTA resin against purified recombinant proteins. GBSS-His_6_ and GST-PTST were detected by immunoblotting with anti-His and anti-GST antibodies respectively. Wild-type GBSS specifically pulls down PTST. Charge-shift mutations at the coiled coil reduce the interaction. (B) Visualisation of GBSS activity in-gel. Recombinant proteins were separated on SDS-PAGE gel containing 0.05% (*w/v*) amylopectin and renatured in-gel. Following incubation with ADP-glucose, activity was visualised by staining with iodine. (C) *In vivo* pulldown experiment by immunoprecipitation of tandem affinity purification (TAP) (c-Myc)-tagged PTST protein transiently co-expressed with variant forms of HA-tagged GBSS in tobacco leaves. Anti-Myc and anti-HA antibodies were used to detect the precipitated PTST and GBSS proteins respectively.

A substantial amount of PTST protein co-eluted with wild-type GBSS, suggesting that the two recombinant proteins interact directly (Fig. 6A). No interaction occurred between free GST and GBSS, indicating that GBSS interacts specifically with PTST (S5 Fig.). Additionally, all of the charge-shift mutations in the GBSS coiled coil helix reduced the interaction *in vitro*. The most substantial reductions occurred when Lys441 and Glu452 were mutated to carry the opposite charge. No interaction was detected in the E452K variant or when charge-shifts were introduced in both Lys441 and Glu452 (the K441E/E452K variant). The chimeric protein that lacked the coiled coil helix altogether also did not interact with PTST. These results not only demonstrate that PTST and GBSS interact directly, but also implicate electrostatic potentials at the coiled coil region as integral to the interaction.

We assessed whether these charge-shift mutations detrimentally affected GBSS protein structure, by in-gel renaturation assays of GBSS activity. A variant carrying the Glu486→Ala mutation was included as an inactive control: the corresponding residue to Glu486 in *E*. *coli* glycogen synthase (Glu377) was previously shown to be essential for catalytic activity [37]. The wild type GBSS protein elongated amylopectin chains in the gel, indicated by dark blue-staining bands with iodine (Fig. 6B). Such a band was not seen in the E486A variant. All charge-shift mutants retained activity, but none could be detected for the chimeric protein.

To investigate the interaction of the Arabidopsis GBSS and PTST proteins *in planta*, we conducted an immunoprecipitation experiment using transiently expressed proteins in tobacco leaves. Prey constructs were made encoding the wild-type GBSS (or its mutated variants) tagged at the C-terminus with cyan fluorescent protein (CFP) and HA tags in tandem. The bait construct encoded PTST with a C-terminal TAP tag, containing c-Myc epitopes. Agrobacteria strains containing bait or prey constructs were co-infiltrated into tobacco leaves and we confirmed that proteins were evenly expressed among different leaves. The PTST-TAP bait protein was successfully immunoprecipitated from extracted soluble proteins using anti-c-Myc beads (Fig. 6C). The wild-type GBSS was only detected in the immunoprecipitate when PTST-TAP was co-expressed, confirming the protein-protein interaction. Consistent with the findings of the *in vitro* pulldown assay, the charge-shift mutations of GBSS at Glu452, or both Lys441 and Glu452, markedly reduced the interaction.

### The CBM48 Domain Is Required for Glucan Binding in PTST

**Fig 7:**
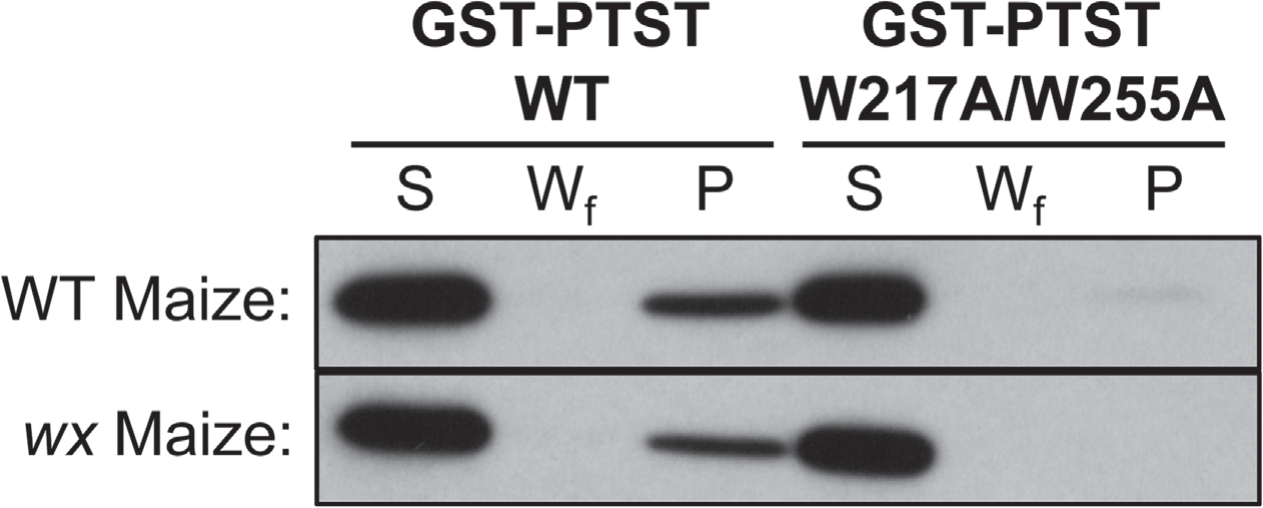
The CBM48 domain is required for glucan-binding in PTST. Binding of GST-PTST recombinant protein to intact wild-type (WT) and waxy (*wx*) maize starch granules was assessed *in vitro*. Unbound proteins are in the soluble fraction (S), while bound proteins are in the pellet (P). No protein was detected in the final wash (W_f_). Mutating both Trp217 and Trp255 in the CBM48 domain abolished the interaction with starch.

A previous study demonstrated that PTST could bind to starch *in vitro* [26]. We compared PTST binding affinity against wild-type and amylose-free *waxy* (*wx*) maize starch granules to test whether it interacted specifically with amylose or amylopectin. Briefly, the GST-PTST recombinant protein was incubated with either wild-type or *wx* starch. The starch was pelleted by centrifugation, and bound proteins were eluted with SDS. We found that PTST could interact equally well with both wild-type and *wx* maize starch (Fig. 7), suggesting that it primarily interacts with amylopectin. Under our assay conditions, free GST did not bind to starch, suggesting that the interaction with starch is via PTST (S6A Fig.). Furthermore, the GST-PTST protein was not found in the pellet fraction when starch was substituted with Sephadex G-10 resin, suggesting that no detectable protein precipitated under these assay conditions. However, the majority of the GST-PTST protein used in the assay remained in the soluble fraction, despite the fact that starch was not limiting in our assay (S6B Fig.). This suggests that binding affinity is low, or that the interaction with starch is transient.

We tested whether the conserved CBM48 domain at the C-terminus of PTST was required for glucan interaction by mutating two conserved tryptophan residues (Trp217 and Trp255) to alanine residues. These residues align with key glucan-binding tryptophans (Trp100 and Trp133) in the CBM48 of the mammalian AMP-kinase β subunit [38]. The W217A/W255A variant of GST-PTST could not interact with either type of starch (Fig. 7), showing that the CBM48 domain is critical for starch binding.

### PTST Is Required for GBSS Localisation on Starch Granules *In Vivo*

The phenotype of Arabidopsis *ptst* mutants strongly suggests that the localisation of GBSS to starch granules requires PTST (Figs. 2–4). To further investigate this, we studied the localisation of fluorescently tagged Arabidopsis PTST and GBSS transiently expressed in tobacco leaves.

**Fig 8:**
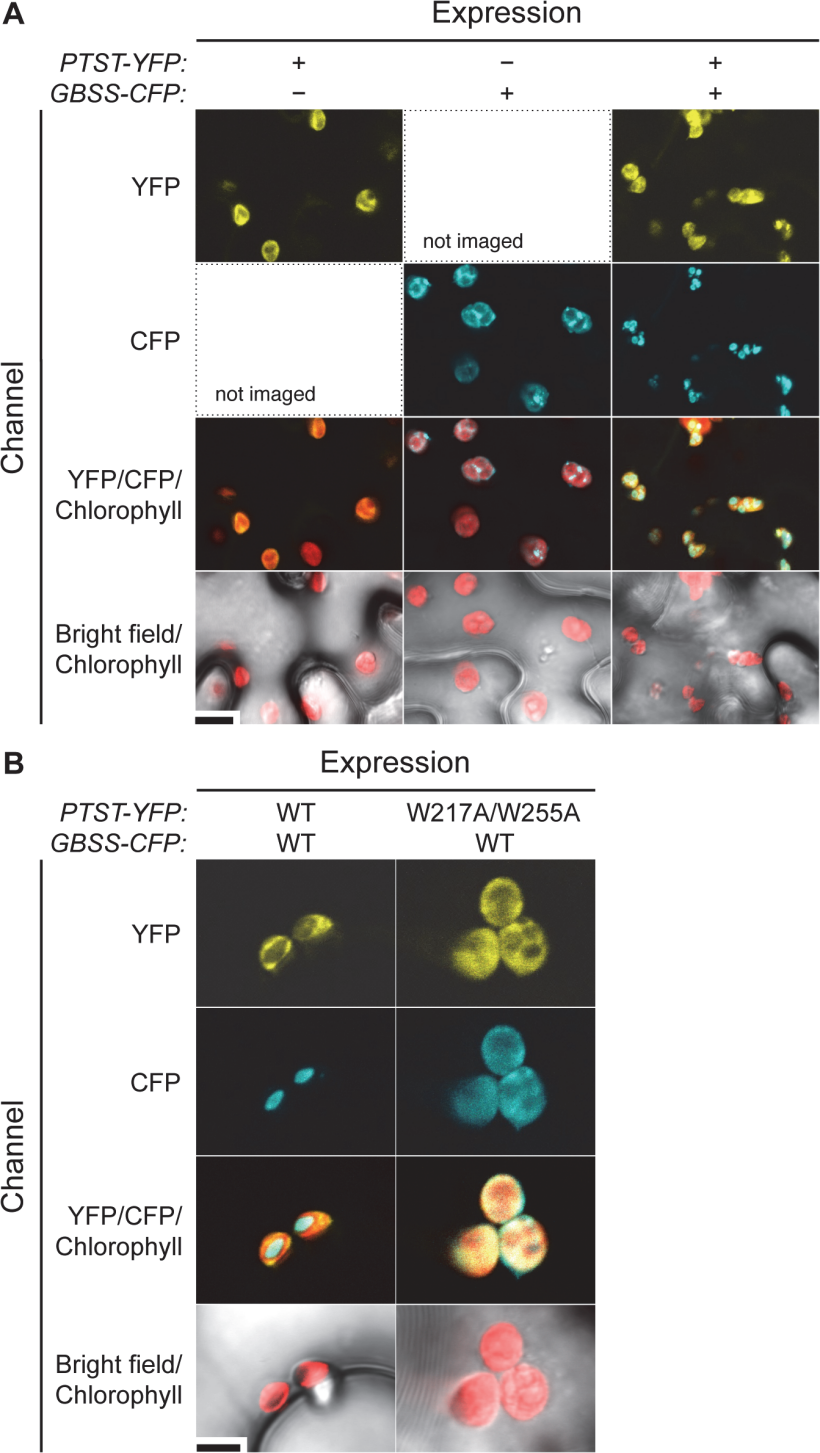
Localisation of fluorescently tagged PTST and GBSS in tobacco leaves. (A) PTST-YFP and GBSS-CFP were individually or co-expressed in tobacco epidermal cells and imaged using confocal microscopy. Note that the localisation of GBSS-CFP on starch granules depends on PTST-YFP co-expression. Bar = 10 μm. (B) Same as (A), but additionally using the W217A/W255A non-starch-binding variant of PTST. Bar = 4 μm.

PTST-yellow fluorescent protein (YFP) and GBSS-CFP proteins were visualised using confocal laser scanning microscopy. When PTST-YFP was expressed alone, the YFP signal was found to co-localise with chlorophyll autofluorescence (Fig. 8A), suggesting that PTST is primarily a free soluble protein in the chloroplast stroma rather than bound to starch granules. This finding is consistent with the abundance of PTST in the soluble fraction of leaf protein extracts (Fig. 3E) and its weak interaction with starch *in vitro* (Fig. 7). Interestingly, a similar stromal localisation was observed for GBSS-CFP when expressed in the absence of the Arabidopsis PTST protein (Fig. 8A). However, when both proteins were co-expressed, PTST-YFP remained stromal while GBSS-CFP localised exclusively to particles within the plastid that are similar in size, shape, and number to starch granules.

The above results provide direct evidence that PTST is required for GBSS localisation onto starch granules in tobacco leaves. However, the fact that PTST remained in the stroma implies that it interacted only transiently with starch during the GBSS localisation. To investigate the functional significance of this transient interaction, we conducted the experiment again with the W217A/W255A variant of the PTST protein, which cannot bind to starch (Fig. 7). We found that GBSS could only localise to the starch granule in the presence of the wild-type PTST-YFP protein, and not in the presence of the W217A/W255A variant (Fig. 8B). These data suggest that the direct interaction between PTST and starch is critical at some point in the GBSS localisation process.

### GBSS Overexpressed in Arabidopsis Requires PTST to Efficiently Localise to Starch

**Fig 9:**
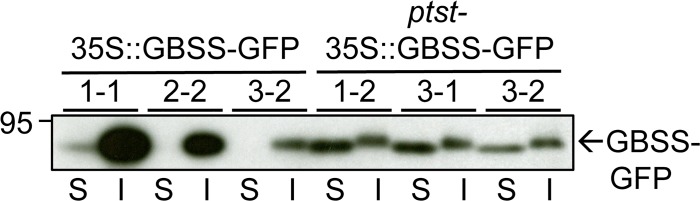
Expression of GBSS-GFP in Arabidopsis *ptst* mutants. Soluble (S) and insoluble (I) protein fractions of leaf extracts were subject to immunoblot analysis with GFP antibodies. Three independent lines generated in both wild-type and *ptst-1* backgrounds are shown, each with varying expression levels. Starch-bound proteins are contained in the insoluble fraction. The small difference in migration between soluble and insoluble fractions was reproducibly observed and may be due to differences in sample preparation and composition.

If PTST is required for GBSS to localise to starch granules, it would be expected that the loss of PTST might cause an accumulation of unbound GBSS in the stroma. However, GBSS was not detected in the soluble fraction of *ptst* mutants (Fig. 3E), most likely because free GBSS is unstable. Therefore, we constitutively overexpressed GFP-tagged Arabidopsis GBSS under the 35S promoter in both wild-type and *ptst-1* plants, isolating multiple independent lines of each. Expression levels of GBSS-GFP varied among transformants. When expressed in wild-type plants (35S::GBSS-GFP lines), we found that GBSS-GFP was almost exclusively in the insoluble fraction (i.e., localised onto starch granules), even in lines where expression was very strong (Fig. 9, lines #1–1, #2–2). In contrast, when GBSS-GFP was overexpressed in *ptst* mutants (*ptst*-35S::GBSS-GFP lines), GBSS-GFP was found primarily in the soluble fraction. This further substantiates the finding that GBSS localisation onto starch granules is inefficient in the absence of PTST. Also, GBSS-GFP protein levels were generally low in *ptst* mutants, and we were not able to isolate lines with levels of expression comparable to the most highly expressing wild-type transformants (e.g., Fig. 9, line #1–1). The lower abundance of GBSS–GFP in the *ptst* background is consistent with the hypothesis that free GBSS is unstable and prone to degradation in the chloroplast stroma.

## Discussion

### A Novel Role for Protein Targeting in Starch Metabolism

Amylose synthesis occurs within a pre-existing matrix of amylopectin in developing starch granules [7,8,39]. Although amylopectin is semi-crystalline, the matrix it forms is hydrated and permeable to small molecules, such as the starch synthase substrate ADP-Glc. The GBSS protein buried within the granule uses ADP-Glc to processively elongate linear chains, which are protected from the activity of branching enzymes in the stroma. Hence, the granule-bound localisation of GBSS is essential for its function. In this study, we have demonstrated that PTST has a previously unknown role in localising GBSS to starch granules. This role is critical for amylose synthesis in Arabidopsis leaves, since mutants lacking PTST accumulate starch containing only amylopectin (Fig. 2C; Table 1).

**Fig 10:**
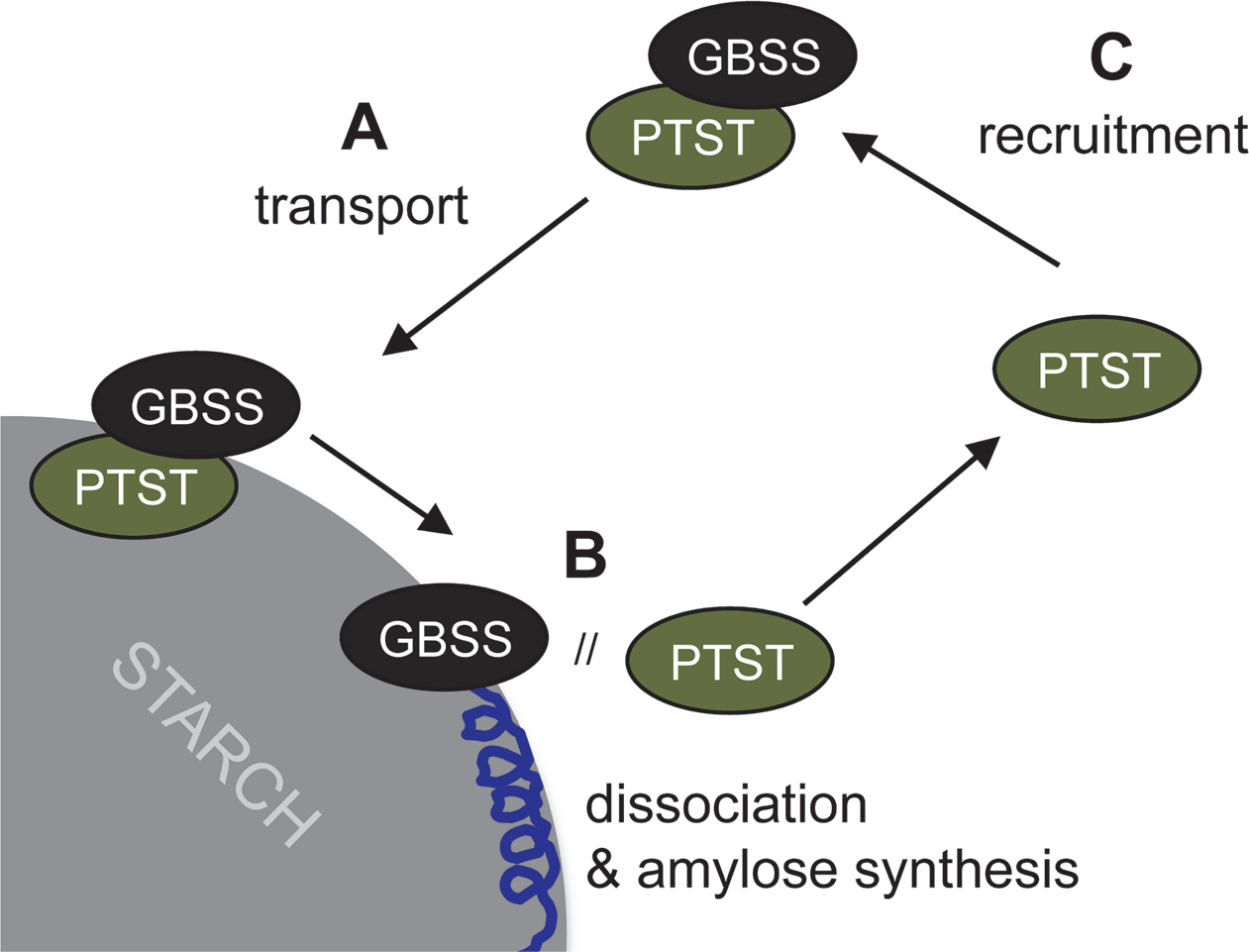
Proposed model of PTST-mediated GBSS localisation to starch. (A) GBSS docks onto PTST in the stroma, and the complex binds to starch. (B) PTST dissociates from both GBSS and the starch granule, leaving GBSS on starch to mediate amylose synthesis. (C) PTST returns to the stroma to recruit another GBSS molecule.

On the basis of our findings, we propose that PTST targets GBSS to the starch granule via a direct transport/delivery mechanism (Fig. 10). In this model, GBSS first interacts with PTST in the chloroplast stroma via the coiled coil helix on the C-terminal domain of GBSS that acts as docking site for PTST (Figs. 5–6). The complex then binds to amylopectin at the surface of the developing starch granules using the CBM48 domain of PTST (Fig. 7). Finally, PTST dissociates both from the granule and from GBSS, leaving GBSS on starch where it can synthesise amylose. Presumably, it becomes buried as amylopectin crystallises above it while PTST returns to the stroma to recruit another GBSS molecule.

We do not fully understand the mechanism behind the final dissociation step. However, there is evidence that this step occurs, as opposed to PTST acting as a permanent “anchor” for GBSS onto the granule. Firstly, GBSS is by far the most abundant protein on Arabidopsis starch, while PTST is far less abundant and only detectable by immunoblotting (Fig. 3A and 3D). Such a difference in amount strongly argues against an anchoring role, where a more even stoichiometry would be expected. Secondly, the localisation of Arabidopsis GBSS onto starch granules in tobacco leaves required the co-expression of Arabidopsis PTST (Fig. 8). If PTST was acting as an anchor for GBSS onto starch, both proteins would localise to the granule. On the contrary, PTST remained in the stroma as GBSS localised to starch granules. Consistent with these localisations, the amount of granule-bound PTST detected in wild-type Arabidopsis in Fig. 3D is small compared to stromal PTST (Fig. 3E). The small amount in the granule probably gets trapped when amylopectin crystallises around it before it dissociates from its GBSS cargo [40,41]. The fact that PTST was not present on starch granules from the *gbss* mutant (Fig. 3D) supports this model, suggesting that PTST does not interact with the starch granule in the absence of GBSS. Indeed, interaction with GBSS may be a pre-requisite for subsequent granule binding.

### Why Is Such a Targeting Mechanism Necessary?

To our knowledge, PTST is the first example of a protein with a dedicated role in targeting other proteins to the starch granule. However, the idea of proteins localising to starch granules via interactions with other proteins has been previously discussed in the literature. It was suggested that SS1 and a branching enzyme (BE2b) in cereal endosperms are brought to the starch granule by forming a complex with SS2, which plays the primary starch-binding role [42]. It is unclear why these proteins, along with GBSS, require such mechanisms to bind to starch efficiently.

While GBSS does not have a specialised carbohydrate-binding module, it has carbohydrate binding residues in the C-terminal domain, which are important for coordinating the substrate in the active site—a shared feature in all starch/glycogen synthases [35]. The presence of these residues may explain the small amount of GBSS detected on the *ptst* starch granules (Fig. 3C), which increased when starch was always present (as seen in the *ptst sex4* double mutant; Fig. 4A). Also, when constitutively over-expressed, some GBSS-GFP protein localised to starch granules in Arabidopsis in the absence of PTST (Fig. 9). GBSS is therefore capable of interacting with starch by itself, but PTST greatly improves the efficiency with which it does so. A further possibility is that PTST delivers GBSS to specific sites at the surface of the developing starch granule, where amylose initiation is most suitable. Any such specificity would likely be defined by the CBM48 domain, which we have shown to play an essential role during the targeting process (Fig. 8B). We are currently characterising the binding specificity of this CBM48 domain compared with other CBMs.

### Specialised Interaction Sites on GBSS Mediate PTST-Docking

Our work suggests that the coiled coil helix on the GBSS C-terminal domain is a PTST interaction site, and that Lys441 and Glu452 are key PTST-binding residues. Mutagenesis of these charged amino acids substantially reduced PTST interaction (Fig. 6). This finding is not surprising since electrostatic interactions between charged amino acids typically play a major role in coiled coil-mediated interactions [29].

However, protein-protein interactions often involve many amino acids forming an interaction surface [43]. While our study implies that the GBSS coiled coil has a central role in forming the interface, other amino acids on adjacent helices may strengthen the interaction. This could explain the residual binding of PTST in some of the charge-shift mutants of GBSS, which was apparent in the immunoprecipitation assay from tobacco leaves, even when no interaction was detected in our *in vitro* assay (Fig. 6A and 6C).

Interestingly, Lys441 and Glu452 are not fully conserved amongst GBSS orthologs (S4A Fig.). On the one hand, their conservation may not have been critical for selection due to the presence of other PTST-binding residues. On the other hand, there may have been co-evolutionary pressures on PTST and GBSS, where mutations on one protein were adjusted for by complementary mutations on the partner protein. A consequence of such co-evolution would be limited cross-species compatibility between PTST and GBSS. Such limited compatibility was recently documented for another complex involved in starch metabolism, the ISA1/ISA2 complex, where partnering the Arabidopsis ISA1 with the rice ISA2 protein could not restore normal levels of activity [44]. In our experiments, Arabidopsis GBSS did not localise onto starch granules in tobacco leaves without the co-expression of Arabidopsis PTST, even though the endogenous tobacco PTST was presumably present (Fig. 8). This could be due to cross-species incompatibility but could also be explained by our use of the 35S promoter for transient overexpression of GBSS. The large amounts of GBSS may have exceeded the capacity of the tobacco PTST, and hence additionally requiring the PTST overexpression efficiently localise all the GBSS to starch granules.

The presence of a coiled coil helix is not specific to GBSS. COILS prediction analysis also suggests that SS2 has a predicted coiled coil in the same region of the protein as GBSS (S3 Data), while SS4 has two predicted coiled coils in its N-terminal domain [45,46]. However, there is no evidence that either SS2 or SS4 functions are compromised by the loss of PTST. Loss of SS2 causes significant changes in the chain length distribution of amylopectin, as well as changes in granule morphology [47,48]. The *ptst* mutants produce amylopectin with a chain length distribution indistinguishable from the wild type (S7A Fig.), and starch granule morphology is unchanged (S7B Fig.). Loss of SS4 also affects granule morphology, but also drastically reduces the granule numbers per chloroplast [49,50]. The Arabidopsis *ss4* mutant phenotype is characterised by the loss of starch in younger leaves, and chloroplasts of older leaves contain one large starch granule. Neither of these phenotypes were observed in the *ptst* mutant (Figs. 2C and S7C). Additionally, zymogram activity patterns of hydrolases, debranching enzymes, and starch synthases in soluble protein extracts from *ptst* mutants were identical to wild type (S7D Fig.). These data suggest that GBSS may be the exclusive interaction partner for PTST, although further work would be necessary to determine this conclusively. In particular, it should be investigated whether the predicted coiled coil on SS2 mediates an interaction with PTST or other proteins. Interestingly, our COILS analysis predicted that the recently identified rice protein FLOURY ENDOSPERM 6 also contains coiled coils (S4 Data). This protein also contains a CBM48 domain and has been shown to influence starch synthesis in rice endosperms, potentially via interaction with isoamylase-type debranching enzymes [51].

### *PTST* Is a Novel Gene Target for Use in Biotechnology to Control Amylose Content

The synthesis of amylose-free starch is a desirable trait in many species and such starches are extensively used by both food and non-food industries [19]. Our study has demonstrated that the *PTST* gene is essential for amylose synthesis in Arabidopsis leaves. This finding was unexpected because, to our knowledge, all amylose-free mutants described in other species are defective in the *GBSS* gene. Extensive screens for amylose-free mutants have been conducted in many cereal crop species, as well as pea, potato, and cassava [13,14,52]. It is therefore surprising that *ptst* mutant alleles have not been isolated in these screens, despite the sequence conservation (and presumably functional conservation) of PTST among higher plants (Fig. 1). One explanation would be the existence of two or more functionally redundant *PTST* paralogues in other species. Alternatively, *ptst* mutants in crop species may synthesise starch with substantial reductions in amylose content, rather than amylose-free starch. Our result with the *ptst sex4* double mutant suggests that GBSS (and amylose) can accumulate slowly in starch granules over extended periods of starch synthesis, even in the absence of PTST (Fig. 4; Table 2). In storage organs, starch is also synthesised over relatively long periods of time (i.e., during seed filling or tuber/storage root development). Furthermore, iodine-based screening methods are excellent for detecting amylose-free phenotypes, since the absence of dark blue amylose-staining can be easily detected by eye [13,14], but poor for detecting low-amylose mutants as the screening method is not quantitative.

It is also worth noting that distinct isoforms of GBSS can mediate amylose synthesis in different tissues [53,54]. In cereal species, there is an endosperm-specific GBSS1 isoform and a leaf-specific GBSS2 isoform [55]. Since Arabidopsis only has the GBSS2-type isoform [55], we cannot conclude from our study whether PTST is required for GBSS1-mediated amylose synthesis in cereal endosperm starches. However, we predict that it is required since the rice PTST gene is strongly expressed in the developing endosperm (S8 Fig.) and GBSS1 isoforms are also predicted to contain a coiled coil helix (S4A Fig.).

Despite the commercial value of manipulating amylose content, GBSS previously represented the only gene target that controls amylose content directly. Our study shows that GBSS is necessary but not sufficient for amylose synthesis in Arabidopsis leaf starch. Therefore PTST represents a new potential target gene, the expression of which may be modified to manipulate amylose content. In Arabidopsis, the loss of PTST altered amylose content with no effect on starch content (Table 1). Thus, reducing PTST expression in other species could reduce the amylose content of starches with no yield penalty. According to previous work, GBSS appears not to catalyse the rate-limiting step in amylose synthesis. Attempts to overexpress the gene in potato and wheat have not resulted in more amylose [56,57]. Our study raises the possibility that PTST may limit GBSS delivery and thereby amylose synthesis. PTST overexpression, particularly in conjunction with GBSS may be an effective strategy to increase amylose content. This may be of interest because currently the production of high-amylose starch is achieved by reducing the expression of branching enzyme [23]. However, this approach incurs yield penalties and also changes amylopectin structure [21,23]. We are currently studying the effect of PTST overexpression in Arabidopsis to further investigate its potential to increase amylose content.

## Materials and Methods

### Plant Materials and Growth Conditions

For all experiments, *A. thaliana* plants were grown on soil in a controlled environment chamber (Percival AR-95L, CLF Plant Climatics; OR Kälte 3000) under a 12-h light/12-h dark cycle. Light intensity was set to 150 μmol photons m^−2^ s^−1^, temperature was set to 20°C, and relative humidity was set to 65%.

The following T-DNA insertion mutants were used in this study: *ptst-1* (SALK_025022; ecotype background Columbia), *ptst-2* (FLAG_622E03; ecotype background: Wassilewskija), *gbss* (GABI_914G01; ecotype background Columbia; [9]), *dpe1-2* (GABI_339B11; ecotype background Columbia; [58]), *sex4-3* (SALK_102567; ecotype background Columbia; [32]). Refer to S1 Table for more details.

### Starch Content and Iodine Staining

Starch was quantified as previously described [59]. Briefly, entire rosettes of 4-week-old plants were homogenised in 0.7 M perchloric acid. The insoluble material was pelleted by centrifugation, washed three times in 80% (*v/v*) ethanol, and resuspended in water. The starch in the insoluble fraction was gelatinised at 95°C for 15 min, and subsequently digested to glucose at 37°C using α-amylase and amyloglucosidase (Roche). Starch content (in glucose equivalents) was determined by quantifying the released glucose with a hexokinase/glucose-6-phosphate dehydrogenase-based spectrophotometric assay.

For iodine staining of plant material, rosettes were harvested and decolourised in 80% (*v/v*) ethanol. Excess ethanol was removed by rinsing in water, before staining in Lugol solution (Sigma-Aldrich). Plants were destained in water for several hours for optimal visualisation of the amylose-iodine complex in starch

### Purification of Starch Granules from Arabidopsis

Entire rosettes of 4-week-old plants were homogenised in starch extraction medium (50 mM Tris-HCl [pH 8], 0.2 mM EDTA and 0.5% [*v/v*] Triton X-100) using a Waring blender. Insoluble material was resuspended in the extraction medium and sequentially filtered through 30 μm and 15 μm nylon nets. Starch granules were separated from the filtrate at 2,500*g* for 15 min over a 95% (*v/v*) Percoll cushion (Sigma-Aldrich). The starch pellet was then washed in 0.5% (*w/v*) SDS. Excess SDS was removed by extensively washing the pellet in water. The pellet was then washed once in 80% (*v/v*) ethanol, and dried under vacuum overnight.

### Analysis of Starch Composition and Structure

The amylose content of the purified starch granules was determined using an iodine colourimetry-based method, as previously described [30]. For separation of amylopectin and amylose on a Sepharose CL-2B column, the following protocol was followed: purified granules (5 mg) were dissolved in 1 M NaOH (0.5 ml), and 0.1 ml was loaded onto a Sepharose CL-2B column (30 cm × 10 mm internal diameter), pre-equilibrated with 50 mM NaOH. Samples were run through the column using 50 mM NaOH as the eluent at a flow rate of 0.2 ml/min. Fractions of 1 ml were collected. The same volume of HCl was used to neutralise each fraction prior to adding Lugol solution to visualise amylose and amylopectin. Absorbance was measured at 595 nm.

For amylopectin chain length distribution, insoluble fractions from perchloric acid-extracted Arabidopsis rosettes were debranched enzymatically, and analysed by high performance anion exchange chromatography with pulsed amperometric detection (HPAEC-PAD) as described previously [44].

For scanning electron microscopy (SEM), purified granules were visualised in a Quanta 250 FEG microscope (FEI).

For light microscopy analysis of starch granules in chloroplasts, leaf segments from 3-week-old plants were fixed in 2% (*v/v*) glutaraldehyde; 0.05 M sodium cacodylate (pH 7.4), for 7 h at 22°C. The segments were washed three times with 0.1 M sodium cacodylate (pH 7.4), and then incubated at 4°C overnight in 1% (*w/v*) osmium tetroxide in 0.1 M sodium cacodylate (pH 7.4). Samples were then washed three times in cold 0.1 M sodium cacodylate (pH 7.4), and once with water. The samples were dehydrated in a series of aqueous ethanol solutions from 50% (*v/v*) to 100% ethanol, then finally with 100% acetone. The segments were embedded in Epon epoxy resin (Fluka). Sections were cut with a diamond knife and stained with a 1% (*w/v*) toluidine blue prior to microscopy. Images were acquired on an AxioImager Z2 microscope fitted with a 100× oil-immersion lens with 1.4 numerical aperture, and AxioCam monochrome camera (Carl Zeiss).

### Protein Extraction Methods

For the extraction of leaf soluble proteins: young leaves were homogenised with a pestle and mortar in protein extraction medium (40 mM Tris-HCl [pH 6.8], 5 mM MgCl_2_, Complete protease inhibitor [Roche]). Insoluble material was pelleted at 20,000*g*, and soluble proteins were collected in the supernatant. Protein content was determined using the Protein Assay kit (Bio-rad), and equal amounts of protein per sample were loaded onto SDS-PAGE gels.

For the fractionation of soluble and insoluble proteins: 7 mm leaf discs were harvested from young leaves at the end of day and snap frozen in liquid N_2_. The disc was homogenised using a pestle and mortar in protein extraction medium and soluble proteins collected in the supernatant as above. Insoluble material in the pellet was washed once in protein extraction medium, and subsequently resuspended in an equivalent volume of SDS-PAGE loading medium (50 mM Tris-HCl [pH 6.8], 2% [*w/v*] SDS, 100 mM DTT, 3% [*v/v*] glycerol, 0.005% [*w/v*] bromophenol blue). The suspension was then heated at 95°C for 5 min and insoluble material was removed by centrifugation. Volumes of soluble protein and insoluble protein extracts corresponding to equal leaf area were loaded onto SDS-PAGE gels.

For the extraction of starch granule-bound proteins: dried purified starch granules were resuspended at a concentration of 33 mg/ml in SDS-PAGE loading medium (see above) and heated at 95°C for 10 min. Gelatinised starch was then pelleted at 20,000*g* for 10 min. Proteins in the supernatant were separated on SDS-PAGE gels. The gels were stained with the Silver Stain Plus kit (Bio-rad).

### Detection of Proteins by Immunoblotting

For immunoblotting, proteins were transferred onto a PVDF membrane following SDS-PAGE. GBSS was detected using polyclonal antibodies raised either against the purified pea GBSS protein [60] or against the purified recombinant Arabidopsis GBSS protein (this study). The PTST protein was detected using polyclonal antibodies raised against either the recombinant His-tagged Arabidopsis PTST protein [26], or against the purified Arabidopsis GST-PTST protein (this study). The GBSS-GFP fusion protein was detected by SDS-PAGE followed by immunoblotting with an anti-GFP antibody (Clontech JL-8). The anti-GST (Abcam) and anti-PentaHis (Qiagen) antibodies were used for the immuno-detection of GST-PTST and GBSS-His_6_ recombinant proteins, respectively.

### Phylogenetic and Bioinformatics Analyses

For building the phylogenetic tree, sequences were retrieved on the NCBI and Phytozome databases using pBLAST. The alignment was constructed using both ClustalW on Bioedit [61] and MAFFT [62]. The best model (JTT+F+G+I) for both alignments was selected using the corrected Akaike Information Criterion (AICc) on the ProtTest Server [63]. Maximum likelihood trees were built using MEGA v6 [64]. Branch support was assessed by performing 500 bootstrap replicates. Both alignments resulted in coherent trees. The alignment used to generate the tree in Fig. 1 can be found in S1 Data. The complete dataset is available from TreeBASE (accession: study 16699).

The predictions of coiled coils were carried out on the COILS/PCOILS server [34]. Results obtained with a 14-amino-acid prediction window are shown. The homology model of the Arabidopsis GBSS protein was generated using the SWISSMODEL server [65]. The amino acid sequence of Arabidopsis GBSS lacking the 79-amino-acid N-terminal chloroplast transit peptide was submitted. The rice GBSS1 crystal structure (pdb: 3VUE; [35]) was selected as a template. The generated model included amino acids 84–587.

### Recombinant Protein Expression in *E*. *coli*

For GST-tagged PTST recombinant protein expression in *E*. *coli*, a PTST-cTP::pGEX-4T-2 IPTG-inducible expression vector was cloned as follows: A full length cDNA clone encoding the PTST protein was obtained from the RIKEN Bioresource Center (http://www.brc.riken.jp; pda04554, RAFL08-09-K08). The region encoding the PTST protein (lacking the predicted 44-amino acid N-terminal chloroplast transit peptide) was amplified with BamHI and NotI restriction sites using the following primers, 5´-ATGGATCCGCTTCTACTCGAAAACATTAC-3´ and 5´-TAGCGGCCGCCTATTCCACCACTAAAACATTG-3´, and cloned into the pGEX-4T-2 vector (GE Healthcare).

For GBSS recombinant protein expression in *E*. *coli*, a GBSS-cTP::pET24a+ IPTG-inducible expression vector was constructed. Arabidopsis cDNA was prepared from wild-type Columbia plants using the RNeasy Plant total RNA extraction kit (Qiagen) and Wizard Reverse transcription kit (Promega). The cDNA region encoding the GBSS protein (lacking the predicted 79-amino acid N-terminal chloroplast transit peptide) was amplified from the cDNA preparation with NheI and XhoI restriction sites using the following primers: 5´-ATGCTAGCTGTGAGAAAGGAATGTCTGTG-3´ and 5´-ATCTCGAGCGGCGTCGCTACGTTCTC-3´. The amplicon was cloned into the pET24a+ vector (Novagen/Merck) in frame with the C-terminal His-tag.

All point mutations were generated using the QuikChange Site-Directed Mutagenesis Kit (Agilent Technologies) according to the manufacturer’s instructions. For the generation of the chimeric GBSS protein, the full length cDNA of Arabidopsis GBSS was synthesised by Biomatik, with the coding sequence of the coiled coil helix (amino acids 440–454) replaced with the Arabidopsis SSI cDNA sequence encoding amino acids 497–511 of *At*SSI.

*E*. *coli* BL21 (DE3) CodonPlus cells (Agilent Technologies) harbouring the expression vector plasmids were cultured in LB medium at 37°C. Protein expression was induced by adding 1 mM IPTG, and grown overnight at 20°C or 18.5°C for GST-PTST and GBSS, respectively. For GST-PTST purification, cells were resuspended in lysis medium (50 mM Tris-HCl [pH 7.5], 300 mM NaCl, 2 mM DTT, 1 mg/ml lysozyme, and Complete Protease Inhibitor Cocktail [Roche]) and lysed with a microfluidiser (Microfluidics). The lysate was subject to centrifugation at 20,000*g* for 10 min, and the supernatant was incubated with Glutathione Sepharose 4B resin (GE Healthcare). The resin was washed five times in triton wash medium (50 mM Tris-HCl [pH 7.5], 300 mM NaCl, 2 mM DTT, 0.5% [*v/v*] Triton X-100), five times in wash medium (50 mM Tris-HCl [pH 7.5], 300 mM NaCl, 2 mM DTT), and subsequently eluted in elution medium (50 mM Tris-HCl [pH 7.5], 300 mM NaCl, 10 mM reduced glutathione). Free GST was also purified as described for GST-PTST from cells harbouring the empty pGEX-4T-2 vector plasmid. For GBSS purification, the recombinant protein was purified from the lysate using Ni^2+^-NTA agarose (Qiagen) using a previously described protocol [66]. Fractions containing GST-PTST, free GST, or GBSS were pooled and stored in 50 mM Tris-HCl (pH 7), 10% (*v/v*) glycerol, and 2 mM DTT at −80°C. Protein concentration was determined using the Bradford assay reagent (Bio-rad).

### Pull-down Assay with Recombinant Proteins

Purified recombinant proteins (1 μg each) were incubated together with Ni^2+^-NTA resin (Qiagen) for 60 min at 20°C in binding medium (50 mM Tris-HCl [pH 7.5], 300 mM NaCl, 10 mM imidazole, 0.1% [*w/v*] BSA). The resin was washed five times in wash medium (50 mM Tris-HCl [pH 7.5], 300 mM NaCl, 20 mM imidazole, 0.1% [*w/v*] BSA, 0.05% [*v/v*] NP-40), before eluting in elution medium (50 mM Tris-HCl [pH 7.5], 300 mM NaCl, 250 mM imidazole). Proteins were detected by SDS-PAGE followed by immunoblotting (see above).

### In-gel GBSS Assay for Recombinant Proteins

GBSS activity was assessed in SDS-PAGE gels following renaturation, using a protocol modified from [67]. The purified recombinant proteins (2.5 μg each) were separated on 10% (*w/v*) SDS-PAGE gels according to standard protocols, except that 0.05% (*w/v*) amylopectin (Fluka) was included in the resolving gel. After electrophoresis, gels were washed in 50 mM Tris-HCl (pH 8.0), 1 mM DTT, 0.1 mM EDTA, and 25% (*v/v*) isopropanol for 1 h, and then further rinsed with 10 mM Tris-HCl (pH 7.5), and 5 mM DTT for 30 minutes. Gels were then incubated in 6 M guanidinium hydrochloride, 100 mM Bicine-KOH (pH 8), 25 mM potassium acetate, 5 mM DTT, and 2 mM MgCl_2_ for 2 h. After rinsing in 100 mM Bicine-KOH (pH 8.0), 25 mM potassium acetate, 2 mM MgCl_2_ for 30 minutes, gels were incubated in starch synthase activity medium (100 mM Bicine-KOH [pH 8.0], 25 mM potassium acetate, 5 mM DTT, 2 mM MgCl_2_, 10% [*v/v*] glycerol, and 0.8 mM ADP-Glucose) for 16 h. Following incubation, gels were stained in Lugol solution. All steps in this protocol were carried out at 20°C.

### Glucan-Binding Assay

Recombinant GST-PTST (1 μg) was incubated in binding medium (50 mM HEPES-NaOH [pH 7.5], 2 mM MgCl_2_, 1 mM DTT, 0.1% [*w/v*] BSA, 0.01% [*v/v*] Triton X-100) for 30 min at 20°C with maize starch (wild-type or waxy; Sigma-Aldrich). The reactions were mixed end-over-end on a spinning wheel during the incubation. The substrates were pelleted at 5,000*g* for 30 s, and unbound proteins were collected in the supernatant. The substrates were then washed three times in binding medium, and bound proteins were eluted in elution medium (50 mM HEPES-NaOH [pH 7.5], 2 mM MgCl_2_, 1 mM DTT, 2% [*w/v*] SDS). Proteins were detected by SDS-PAGE followed by immunoblotting (see above).

### Transient Expression of Fusion Proteins in Tobacco

The full coding sequence for Arabidopsis PTST was amplified from the cDNA clone described above with the following primers: 5´-ATGGGATGTGTACCCAGAATTG-3´ and 5´-TTCCACCACTAAAACATTGTTCTC-3´, and cloned into the Gateway-compatible entry vector, pCR8 using the pCR8/GW/TOPO TA kit (Invitrogen). The PTST and GBSS constructs were recombined into the Gateway destination vectors, pB7YWG2.0 (YFP; [68]) and pEarleyGate102 (CFP/HA; [69]) respectively, in frame with the C-terminal fluorescent protein tags.

Expression constructs were transformed into *Agrobacterium tumefaciens* (strain GV3101), and cultures were grown at 28°C in LB medium supplemented with the appropriate antibiotics for 22–24 h. Cells were pelleted at 5,000*g* for 15 min and resuspended in water to an OD_600_ of 1. The cell suspension was then infiltrated into the intercellular spaces between abaxial epidermal cells of intact *Nicotiana benthamiana* leaves using a 1 ml plastic syringe. Infiltration of 0.2 ml into each spot yielded approx. 5 cm^2^ coverage of leaf area. After infiltration, plants were placed away from strong light for 48 h.

The plants were placed under illumination for 12 h prior to microscopy. Images were acquired on a Zeiss LSM 780 confocal microscope (Carl Zeiss), with a 40× water-immersion lens with a 1.1 numerical aperture. YFP signals were monitored using an argon laser at 514 nm wavelength for excitation, and emitted light was captured between a wavelength window of 518 to 557 nm. CFP signals were monitored using an argon laser at 458 nm for excitation, and emitted light was captured between 462 to 500 nm. Autofluorescence of chlorophyll was monitored using an argon laser at 514 nm for excitation, and emitted light was captured between 662 to 721 nm. Images were processed using ImageJ software (http://rsbweb.nih.gov/ij/).

### Generation of Transgenic GBSS Overexpression Lines

The full-length coding sequence of the Arabidopsis GBSS protein was amplified from an Arabidopsis cDNA preparation (described above), with the following primers: 5´-ATGGCAACTGTGACTGCTTCTTCTAA-3´ and 5´-CGGCGTCGCTACGTTCTC-3´. The amplicon was cloned into the Gateway-compatible entry vector, pCR8 using the pCR8/GW/TOPO TA kit (Invitrogen). The construct was recombined into the Gateway-compatible pEarleyGate103 vector [69] in frame with the C-terminal GFP/His tag. This vector was introduced into Agrobacterium cells (strain: GV3101), and then transformed into wild-type Columbia and *ptst-1* plants as described previously [70]. Screening was based on resistance to the herbicide Basta. The analyses presented in this work were carried out on resistant T2 seedlings.

### Zymograms

Zymograms on native PAGE gels containing 0.1% (*w/v*) amylopectin and 0.1% (*w/v*) β-limit dextrin were conducted as described previously [66]. Starch synthase zymograms were produced using the same method, except gels contained 0.15% (*w/v*) amylopectin, and the gels were incubated overnight in the starch synthase activity medium (described above for “In-gel GBSS assay for recombinant proteins”). Activity was visualised by staining the gel in Lugol’s solution, followed by extensive destaining in water.

## Supporting Information

S1 Data Sequence alignment used to generate the phylogenetic tree in Fig. 1. (TXT)

S2 Data Quantification of *GBSS* transcripts in the *ptst* mutant using quantitative PCR. (XLSX)

S3 Data Coiled coil predictions on Arabidopsis starch synthase protein sequences. (XLSX)

S4 Data Coiled coil prediction on the Rice FLO6 protein sequence. (XLSX)

S5 Data Numerical data for plots shown in S1, S2, S3, S7A, and S8 Fig. (XLSX)

S6 Data Sequence alignments used to generate panels in S4 Fig. (DOCX)

S1 Fig Starch content of *ptst* mutants over a 24-h day/night cycle. Plants were grown under a 12-h light (0–12 h) and 12-h dark (12–24 h) regime. Entire Arabidopsis rosettes were harvested every 4 h and starch was quantified. Values are the mean ± standard error of the mean (SEM) from 4–5 plants. Note that values at the 0-h time point are replotted from the 24-h time point. No significant differences between mutants and wild type (Col) at *p* < 0.05 were observed at any time point. Numerical data used to generate the plot are provided in S5 Data.
(TIF)

S2 Fig Chromatographic separation of amylopectin and amylose from Arabidopsis starch granules. Purified granules were dissolved in 0.5 M NaOH and separated over a Sepharose CL-2B column. Fractions were mixed with an iodine solution, and absorbance was recorded at 595 nm. (A) Wild-type granules (Ws) versus *ptst-2* starch granules. (B) Wild-type granules (Col) versus *sex4*, *ptst sex4*, and *gbss sex4* starch granules. Numerical data used to generate the plots are provided in S5 Data.
(TIF)

S3 Fig Immunoblot-based quantification of GBSS content in *ptst* starch granules relative to wild type. Granule-bound proteins extracted from purified Arabidopsis *ptst* starch granules, and a dilution series of proteins from wild-type starch, were separated by SDS-PAGE. GBSS was detected by immunoblotting using a GBSS-specific antiserum. The equivalent mass of starch loaded is indicated above each lane, and the corresponding band intensity calculated using ImageJ densitometry software is indicated below. Numerical data used to generate the plot (band intensity) are provided in S5 Data.
(TIF)

S4 Fig The coiled coil is conserved in GBSS sequences from higher plants. All alignments were generated using ClustalW, and the region surrounding the predicted coiled coil of GBSS is shown. (A). Sequence alignment of GBSS sequences from representative species of Viridiplantae. The COILS prediction scores for each individual sequence are indicated below the sequence. Amino acid positions containing Lys441, Glu449, and Glu452 are highlighted in yellow. (B) Generation of the chimeric protein between GBSS and SS1, which does not have a predicted coiled coil. The top panel shows the sequence alignment for *A*. *thaliana* GBSS and SS1 proteins, together with barley SS1 (*Hv*SS1) and rice GBSS1 (*Os*GBSS1) proteins. Structural features from the solved *Hv*SS1 and *Os*GBSS1 structures are depicted below the respective sequences, where helices (h) are represented in blue and sheets (s) are represented in orange. The bottom panel shows the sequence of the chimeric GBSS protein, where the helix containing the coiled coil from *At*GBSS has been swapped with the homologous helix on *At*SS1, together with an indication of COILS prediction scores. The swapped regions are highlighted in yellow. Full alignments used to generate these figures are provided in S6 Data.
(TIF)

S5 Fig The *in vitro* pulldown assay detects interaction between GBSS and PTST specifically. Purified recombinant proteins (1 μg each; Input) were incubated together with a Ni^2+^-NTA resin. Unbound proteins were removed in the supernatant (Flowthrough), and the resin was washed five times. Bound proteins were eluted twice with high imidazole. Proteins were detected by immunoblotting with anti-His for GBSS-His_6_, and anti-GST for GST-PTST, and free GST.
(TIF)

S6 Fig Optimisation of the starch binding assay. (A) Purified GST-PTST, the W217A/W255A variant, and free GST (1 μg each) was incubated with wild-type (WT) maize starch, or Sephadex G-10 as a non-glucan control. After centrifugation, unbound proteins were collected in the soluble fraction. After three washes, bound proteins were eluted from the starch pellet. Protein was detected in the soluble (S) and pellet (P) fractions, as well as the initial and final washes (W_i_ and W_f_) by immunoblotting with anti-GST. (B) The indicated amounts of recombinant GST-PTST protein were incubated with wild-type (WT) or waxy (*wx*) maize starch. Starch binding assay was carried out as described for (A). Unbound proteins were detected in the supernatant (Soluble), while bound proteins were detected in the pellet fraction (Pellet). The amount of bound proteins increased as the amount of protein used in the assay increased. Starch was therefore not limiting for the amounts of protein tested.
(TIF)

S7 Fig The loss PTST affects amylose and GBSS exclusively. (A) Amylopectin chain length distribution is not affected by the loss of PTST. Starch was debranched enzymatically and prior to analysis with high performance anion exchange chromatography (HPAEC-PAD). The area of individual peaks corresponding to chains with a given degree of polymerisation (DP) was expressed as a percentage relative to the summed peak area for DP 2–50. Values represent mean ± standard error of four biological replicates. No significant differences were detected at *p* < 0.05 between wild-type and *ptst* mutant at any DP. Numerical data used to generate the plot are provided in S5 Data. (B) *ptst* mutants produce starch with normal granule morphology. Starch granules were purified from Arabidopsis rosettes and examined under a scanning electron microscope (SEM). Bar = 5 μm. (C) The number of starch granules per chloroplast is not affected by the loss of PTST. Resin-embedded leaf tissue was sectioned and stained with toluidine blue prior to light microscopy. Bar = 10 μm. (D) Zymogram analysis of soluble enzymatic activities in the *ptst* and *gbss* mutants. Soluble protein extracts of Arabidopsis leaves were separated on native PAGE gels containing 0.1% (*w/v*) amylopectin for visualising hydrolytic activities, 0.1% (*w/v*) β-limit dextrin for visualising debranching enzyme activities, and 0.15% (*w/v*) amylopectin for starch synthase activities. Equal amounts of protein were loaded per lane (30 μg). Following electrophoresis, gels were incubated in activity medium, and bands were observed by staining with iodine solution. The starch synthase activity gel was extensively destained in water. No differences were observed in the banding pattern between mutants and wild type (Col).
(TIF)

S8 Fig Expression of GBSS1, GBSS2, and PTST in different organs of rice. Data was obtained from the Genevestigator database, consisting of curated Rice Genome 51K array experiments. As expected, GBSS1 was mainly expressed in the endosperm, while GBSS2 was mainly expressed in leaf tissue. PTST was expressed in both leaf and endosperm tissues. Numerical data used to generate the plot are provided in S5 Data.
(TIF)

S1 Table T-DNA insertion lines used in this study. (DOCX)

## Abbreviations:

CBM48: family 48 carbohydrate binding module
CFP: cyan fluorescent protein
GBSS: GRANULE-BOUND STARCH SYNTHASE
PTST: PROTEIN TARGETING TO STARCH
SEM: scanning electron microscopy
TAP: tandem affinity purification
YFP: yellow fluorescent protein
