# Supplementary material for: PROTEIN TARGETING TO STARCH Is Required for Localising GRANULE-BOUND STARCH SYNTHASE to Starch Granules and for Normal Amylose Synthesis in Arabidopsis
Source: PLoS Biol. 2015 Feb 24;13(2):e1002080. doi: 10.1371/journal.pbio.1002080 (PMC4339375; doi:10.1371/journal.pbio.1002080)
Supplement: S6 Data — (DOCX) [file pbio.1002080.s006.docx]

Full sequence alignment used to produce Fig S4A
(region shown in figure highlighted yellow)

CLUSTAL 2.1 multiple sequence alignment

HordeumvulgareGBSSI_AHC55210.1 MAALATSQLATSGTVLGVTDR-----FRRPGFQGLRPRNPA--DAALGMR 43

OryzasativaGBSSI_AAF72562.1_ MSALTTSQLATSATGFGIADRSAPSSLLRHGFQGLKPRSPAGGDATSLSV 50

ZeamaysGBSSI_NP_001105001_ MAALATSQLVATRAGLGVPDAS---TFRRGAAQGLR-GARASAAADTLSM 46

HordeumvulgareGBSSII_BAJ99426. ------------MGSYQTNSVGSLKLSPHIQFQQSCNNGVMFLS-----M 33

ZeamaysGBSSII_NP_001106039.1_ MAATMGSI--SANGSYQTNRPSALKQAPHMQFQQCCNGGLRFLSKHSQSM 48

OryzasativaGBSSII_AAL58572.1_ MARTMGST--PTYCSYQTNGVGALKQSPHMQFQQSYNYGVRFLKRDTLSV 48

ManihotesculentaGBSS_AET43458. MATVIAAH-LVSRSSHLSIH---ALETKANNLSHTGPWT-QTITPNGLRS 45

Eucalyptusgrandis_KCW72603.1_ MATITAAH-FVSRSSQVNCHGSTGAEAKVN-LAQISLRN-ASITHSGLRS 47

PrunuspersicaGBSSII-1_AFA36447 MSTLAASH-FVSRTSHVNGG--SGSETRTN-LGQMGLWN-QPMTHNGLRS 45

SolanumtuberosumGBSS_ABY89288. MASITASHHFVSRSQ-------TSLDTKST-LSQIGLRN-HTLTHNGLRA 41

ArabidopsisthalianaGBSS_AAM744 MATVTASSNFVSRTSLFNNHG----ASSCSDVAQITLKG-QSLTHCGLRS 45

PrunuspersicaGBSSII-2_AFA36448 MATVTASS-FASTSSNVSYGAVSSVSEPKTGYKKMGFGK-QAMTHNGLRA 48

Vitisvinifera_XP_002273608.1_ MATVTASN-FLPRSSNGNCGATCRIEKKAT-LTKLRPKI-QILAHNGLRA 47

PisumsativumGBSSIIb_CAC69955.1 MATVTASSNIVSRTSHVNLPTVSCEFKTAP--MRLGSIR-KANTHNGLRV 47

PisumsativumGBSSIIa_AAB26591.1 MATITGSS-MPTRTACFNYQG-RSAESKLN-LPQIHFNNNQAFPVLGLRS 47

Chlamydomonasreinhardtii_AAC17 MAVASTSR----------------PSSARPIVINAASFGVKKTANQLLRE 34

HordeumvulgareGBSSI_AHC55210.1 TIGASAAPKQSRKAHRGSRRCLS-----------VVVSATGSGMNLVFVG 82

OryzasativaGBSSI_AAF72562.1_ TTSARATPKQQRSVQRGSRRFPS-----------VVVYATGAGMNVVFVG 89

ZeamaysGBSSI_NP_001105001_ RTSARAAPRHQQQARRGGR-FPS-----------LVVCAS-AGMNVVFVG 83

HordeumvulgareGBSSII_BAJ99426. RNKTQLAKRRATNYETHRNSSRT-----------SSPIVCSTGMPIIFVA 72

ZeamaysGBSSII_NP_001106039.1_ RSKIQVAKRRATDNGIHPKTTGH-----------RAPIVCSAGMTIVFVA 87

OryzasativaGBSSII_AAL58572.1_ RINKHMAKRIATSTGICTKPRRS-----------HMPIVCSAGMTIIFIA 87

ManihotesculentaGBSS_AET43458. LNTMDKLQMKTQS-----KAVKK--VSATGNGRPAAKIICGHGMNLIFVG 88

Eucalyptusgrandis_KCW72603.1_ VNKLDALRSHAKAAKG--KVERK--VNKAENDGLAGKIVCRKGMNLIFVG 93

PrunuspersicaGBSSII-1_AFA36447 LSNLDMLRIKTRPNAVPRQAMKK--ADKTEGDQCVGKIVCGTGMNLVFVG 93

SolanumtuberosumGBSS_ABY89288. VNKLDGLQSRTNTKVTPKMAFRT--ETKRPG--CSATIVCGKGMNLIFVG 87

ArabidopsisthalianaGBSS_AAM744 FNMVDNLQRRSQAKPVSAKSSKR--SSKVKT---AGKIVCEKGMSVIFIG 90

PrunuspersicaGBSSII-2_AFA36448 LNTVDELRVKIMGNSIARQARSKSFNSTRTGSRPAGTIVCGSGMNLVFLG 98

Vitisvinifera_XP_002273608.1_ LNSVDELHQRTT-IKVTRKGSRKGFKNE--NPRPWGGIICGCGMSLIFVG 94

PisumsativumGBSSIIb_CAC69955.1 LNSLDELLNRTPIKMKGVQSRKKGVQRK--NVRPKGIIVCG--MNLIFVG 93

PisumsativumGBSSIIa_AAB26591.1 LNKLHVRTARATSGSSDTSEK------------SLGKIVCG--MSLVFVG 83

Chlamydomonasreinhardtii_AAC17 LARGSARKSTSRSAVTGATGATC-------------------ALDIVMVA 65

: ::::.

HordeumvulgareGBSSI_AHC55210.1 AEMAPWSKTGGLGDVLGGLPPAMAANGHRVMVVSPRYDQYKDAWDTSVIS 132

OryzasativaGBSSI_AAF72562.1_ AEMAPWSKTGGLGDVLGGLPPAMAANGHRVMVISPRYDQYKDAWDTSVVA 139

ZeamaysGBSSI_NP_001105001_ AEMAPWSKTGGLGDVLGGLPPAMAANGHRVMVVSPRYDQYKDAWDTSVVS 133

HordeumvulgareGBSSII_BAJ99426. TEVHPWCKTGGLGDVVGGLPPALAAMGHRVMTIAPRYDQYKDTWDTNVLV 122

ZeamaysGBSSII_NP_001106039.1_ TEVHPWCKTGGLGDVVGGLPPALAAMGHRVMTIAPRYDQYKDAWDTSVLV 137

OryzasativaGBSSII_AAL58572.1_ TECHPWCKTGGLGDVLGGLPPALAAMGHRVMTIVPRYDQYKDAWDTNVLV 137

ManihotesculentaGBSS_AET43458. AEVGPWSKTGGLGDVLGGLPPAMAARGHRVMTVSPRYDQYKDAWDTSVSV 138

Eucalyptusgrandis_KCW72603.1_ AEVAPWSKTGGLGDVLGGLPPAMAARGHRVMTVSPRYDQYKDGWDTSVLA 143

PrunuspersicaGBSSII-1_AFA36447 AEVGPWSKTGGLGDVLGGLPPALAANGHRVMTVSPRYDQYKDAWDTSVLV 143

SolanumtuberosumGBSS_ABY89288. TEVGPWSETGGLGDVLGGLPPALAARGHRVMTMSPRYDQYKDTWDTSVAV 137

ArabidopsisthalianaGBSS_AAM744 AEVGPWSKTGGLGDVLGGLPPALAARGHRVMTICPRYDQYKDAWDTCVVV 140

PrunuspersicaGBSSII-2_AFA36448 TEVGPWSKTGGLGDVLGGLPPAMAANGHRVMTVSPRYDQYKDAWDTEVTI 148

Vitisvinifera_XP_002273608.1_ TEVGPWSKTGGLGDVLGGLPPAMSANGHRVMTVSPRFDQYKDAWDTGVTV 144

PisumsativumGBSSIIb_CAC69955.1 TEVAPWSKTGGLGDVLGGLPPALSANGHRVMTVTPRYDQYKDAWDTNVTI 143

PisumsativumGBSSIIa_AAB26591.1 AEVGPWSKTGGLGDVLGGLPPVLAGNGHRVMTVSPRYDQYKDAWDTNVLV 133

Chlamydomonasreinhardtii_AAC17 AEVAPWSKTGGLGDVTGGLPIELVKRGHRVMTIAPRYDQYADAWDTSVVV 115

:* **.:******* **** : *****.: **:*** * *** *

HordeumvulgareGBSSI_AHC55210.1 EIKVADEYERVRFFHCYKRGVDRVFIDHPWFLEKVRGKTKEKIYGPDAGT 182

OryzasativaGBSSI_AAF72562.1_ EIKVADRYERVRFFHCYKRGVDRVFIDHPSFLEKVWGKTGEKIYGPDTGV 189

ZeamaysGBSSI_NP_001105001_ EIKMGDGYETVRFFHCYKRGVDRVFVDHPLFLERVWGKTEEKIYGPVAGT 183

HordeumvulgareGBSSII_BAJ99426. EVIVGDRTETVRFFHCYKRGVDRVFVDHPMFLEKVWGKTGSKLYGPTTGT 172

ZeamaysGBSSII_NP_001106039.1_ EVNIGDTVETVRFFHCYKRGVDRVFVDHPMFLEKVWGKTGAKLCGPTTGT 187

OryzasativaGBSSII_AAL58572.1_ EVNIGDRTETVRFFHCYKRGVDRVFVDHPMFLEKVWGKTGPKLYGPTTGD 187

ManihotesculentaGBSS_AET43458. EIKIGDRIETVRFFHSYKRGVDRVFVDHPMFLEKVWGKTGSKIYGPRAGL 188

Eucalyptusgrandis_KCW72603.1_ EIKVGDRIETVRFFHCYKRGVDRVFVDHPMFLEKVWGKTGSKVYGPRAGL 193

PrunuspersicaGBSSII-1_AFA36447 EIEVDGKIETVRFFHCYKRGVDRVFVDHPLFLERVWGKTGSKIYGPKTGE 193

SolanumtuberosumGBSS_ABY89288. EVKVGDSIEIVRFFHCYKRGVDRVFVDHPMFLEKVWGKTGSKIYGPKAGL 187

ArabidopsisthalianaGBSS_AAM744 QIKVGDKVENVRFFHCYKRGVDRVFVDHPIFLAKVVGKTGSKIYGPITGV 190

PrunuspersicaGBSSII-2_AFA36448 EVKVGEKTEKVRFFHCYKRGVDRVFVDHPLFLEKVWGKTASKIYGPIAGE 198

Vitisvinifera_XP_002273608.1_ QLNVGDRFEKVRFFHCYKRGVDRVFVDHPWFLEKVWGKTGSKLYGPLAGE 194

PisumsativumGBSSIIb_CAC69955.1 EVKVGDRTEKVRFFHCFKRGVDRVFVDHPIFLEKVWGKTGTKLYGPAAGD 193

PisumsativumGBSSIIa_AAB26591.1 EVKVGDKIETVRFFHCYKRGVDRVFVDHPLFLERVWGKTGSKLYGPKTGI 183

Chlamydomonasreinhardtii_AAC17 DIMG----EKVRYFHSIKKGVHRVWIDHPWFLAKVWGKTGSKLYGPRSGA 161

:: * **:**. *:**.**::*** ** :* *** *: ** :*

HordeumvulgareGBSSI_AHC55210.1 DYEDNQQRFSLLCQAALEAPRILNLNNNPYFSGPYGEDVVFVCNDWHTGL 232

OryzasativaGBSSI_AAF72562.1_ DYKDNQMRFSLLCQAALEAPRILNLNNNPYFKGTYGEDVVFVCNDWHTGP 239

ZeamaysGBSSI_NP_001105001_ DYRDNQLRFSLLCQAALEAPRILSLNNNPYFSGPYGEDVVFVCNDWHTGP 233

HordeumvulgareGBSSII_BAJ99426. DFRDNQLRFCLLCLAALEAPRVLNLNNSEYFSGPYGENVVFVANDWHTAV 222

ZeamaysGBSSII_NP_001106039.1_ DYRDNQLRFCLLCLATLEAPRVLNFNNSEYFSGPYGEDVVFVANDWHTAI 237

OryzasativaGBSSII_AAL58572.1_ DYRDNQLRFCLLCLAALEAPRVLNLNNSEYFSGPYGENVVFVANDWHTGV 237

ManihotesculentaGBSS_AET43458. DYQDNQLRFSLLCLAALEAPRVLNLNSSKNFSGPYGEEVAFIANDWHTAL 238

Eucalyptusgrandis_KCW72603.1_ DYKDNQLRFSLLCQAALEAPRVLNLNNGENFSGPYGEDVVFVANDWHTAL 243

PrunuspersicaGBSSII-1_AFA36447 DYKDNQLRFSLLCQAALEAPRVLNLNSNKYFSGPYGEDVVFVANDWHTAL 243

SolanumtuberosumGBSS_ABY89288. DYLDNELRFSLLCQAALEAPKVLNLNSSNYFSGPYGEDVLFIANDWHTAL 237

ArabidopsisthalianaGBSS_AAM744 DYNDNQLRFSLLCQAALEAPQVLNLNSSKYFSGPYGEDVVFVANDWHTAL 240

PrunuspersicaGBSSII-2_AFA36448 DFKDNQLRFSLLCRAALEAPRVLNLNSSKYFSGPYGEEVVFIANDWHTAL 248

Vitisvinifera_XP_002273608.1_ DFQDNQLRFSLLCQAALEAPRILNLKSSKYFSGPYGEDVVFIANDWHTAP 244

PisumsativumGBSSIIb_CAC69955.1 DYQDNQLRFSIFCQAALEAARVLNLKSNKYFSGPYGEDVIFVANDWHTAL 243

PisumsativumGBSSIIa_AAB26591.1 DYRDNQLRFSLLCQAALEAPRVLNLNSSKYFSGPYGEDVIFVANDWHSAL 233

Chlamydomonasreinhardtii_AAC17 DYLDNHKRFALFCKAAIEAARVLPFGP--------GEDCVFVANDWHSAL 203

*: **. **.::* *::**.::* : **: *:.****:.

HordeumvulgareGBSSI_AHC55210.1 LACYLKSNYQSNGIYRTAKVAFCIHNISYQGRFSFDDFAQLNLPDRFKSS 282

OryzasativaGBSSI_AAF72562.1_ LASYLKNNYQPNGIYRNAKVAFCIHNISYQGRFAFEDYPELNLSERFRSS 289

ZeamaysGBSSI_NP_001105001_ LSCYLKSNYQSHGIYRDAKTAFCIHNISYQGRFAFSDYPELNLPERFKSS 283

HordeumvulgareGBSSII_BAJ99426. LPCYLKSMYKQNGIYENAKVAFCIHNIAYQGRFPRADFELLNLPESFMPS 272

ZeamaysGBSSII_NP_001106039.1_ LPCYLKSMYKPNGIYKNAKVAFCIHNIAYQGRFARADFDLLNLPDSFLPS 287

OryzasativaGBSSII_AAL58572.1_ LPCYLKSIYQAKGMYVNAKVAFCIHNIAYQGRFAREDFELLNLPDSFLPS 287

ManihotesculentaGBSS_AET43458. LPCYLKAIYQPMGIYKHAKVAFCIHNIAYQGRFAFSDFPRLNLPDKFKSS 288

Eucalyptusgrandis_KCW72603.1_ LPCYLKTMYQSRGLYKNAKVAFCIHNIAYQGRFPFGDFALVNLPNEFKSS 293

PrunuspersicaGBSSII-1_AFA36447 IPCYLKTIYKPRGLYKNAKVVFCIHNIAYQGRFSFSDFSLLDLPDHLRGS 293

SolanumtuberosumGBSS_ABY89288. IPCYLKSMYQSRGIYLNAKVAFCIHNIAYQGRFSFSDFPLLNLPDEFRGS 287

ArabidopsisthalianaGBSS_AAM744 LPCYLKSMYQSRGVYMNAKVVFCIHNIAYQGRFAFDDYSLLNLPISFKSS 290

PrunuspersicaGBSSII-2_AFA36448 LPCYLKAIYQPKGIYRSAKVAFCIHNIAYQGRFASADFALLNLPDEFKSS 298

Vitisvinifera_XP_002273608.1_ LACYLKTMYRLKGRYGNAKVVFCIHNIAYQGRFAFADFSLLNLPDEFKSS 294

PisumsativumGBSSIIb_CAC69955.1 ISCYMKSMYQSIGIFRNAKVVFCIHNIAYQGRFAFTDYSLLNLPDQFKSS 293

PisumsativumGBSSIIa_AAB26591.1 IPCYLKSMYKSRGLYKNAKVAFCIHNIAYQGRNAFSDFSLLNLPDEFRSS 283

Chlamydomonasreinhardtii_AAC17 VPVLLKDEYQPKGQFTKAKSVLAIHNIAFQGRMWEEAFKDTKLPQAAFDK 253

:. :* *: * : ** .:.****::*** : .*. .

HordeumvulgareGBSSI_AHC55210.1 FDFIDGYDK--------------PVEGR---KINWMKAGILQADKVLTVS 315

OryzasativaGBSSI_AAF72562.1_ FDFIDGYDT--------------PVEGR---KINWMKAGILEADRVLTVS 322

ZeamaysGBSSI_NP_001105001_ FDFIDGYEK--------------PVEGR---KINWMKAGILEADRVLTVS 316

HordeumvulgareGBSSII_BAJ99426. FDFVDGHVK--------------PVVGR---KINWMKAGITECDVVLTVS 305

ZeamaysGBSSII_NP_001106039.1_ FDFIDGHVK--------------PVLGR---KLNWMKAGIIESDMVLTVS 320

OryzasativaGBSSII_AAL58572.1_ FDFIDGHFK--------------PVVGR---KINWMKAGITECDLVMTVS 320

ManihotesculentaGBSS_AET43458. FDFIDGYEK--------------PVKGR---KINWMKAGILESDRVLTVS 321

Eucalyptusgrandis_KCW72603.1_ FDFIDGNLK--------------PAKGR---KINWMKAGIIESHRVLTVS 326

PrunuspersicaGBSSII-1_AFA36447 FDFIDGYDK--------------PVKGR---KINWMKAGILESDRVVTVS 326

SolanumtuberosumGBSS_ABY89288. FDFIDGYEK--------------PVKGR---KINWMKAGILESHRVVTVS 320

ArabidopsisthalianaGBSS_AAM744 FDFMDGYEK--------------PVKGR---KINWMKAAILEAHRVLTVS 323

PrunuspersicaGBSSII-2_AFA36448 FDFIDGYDK--------------PVKGR---KINWMKAGILESDKVLTVS 331

Vitisvinifera_XP_002273608.1_ FDFTDGYEK--------------PVKGR---KINWMKAGILEADKVLTVS 327

PisumsativumGBSSIIb_CAC69955.1 FDFLDGHVK--------------PIVGR---KINWMKAGIIESHRVLTVS 326

PisumsativumGBSSIIa_AAB26591.1 FDFIDGYNK--------------PCEGK---KINWMKAGILESDQVFTVS 316

Chlamydomonasreinhardtii_AAC17 LAFSDGYAKVYTEATPMEEDEKPPLTGKTYKKINWLKGGIIAADKLVTVS 303

: * ** . * *: *:**:*..* .. :.***

HordeumvulgareGBSSI_AHC55210.1 PYYAEELISGEARGCELDNIMR-LTGITGIVNGMDVSEWDPTKDKFLAVN 364

OryzasativaGBSSI_AAF72562.1_ PYYAEELISGIARGCELDNIMR-LTGITGIVNGMDVSEWDPSKDKYITAK 371

ZeamaysGBSSI_NP_001105001_ PYYAEELISGIARGCELDNIMR-LTGITGIVNGMDVSEWDPSRDKYIAVK 365

HordeumvulgareGBSSII_BAJ99426. PHYVKELTSGPEKGVELDGVLRTKPLETGIVNGMDVIDWNPATDKYISVK 355

ZeamaysGBSSII_NP_001106039.1_ PHYVKELISGPDKGVELDGVLRTKPLEIGIVNGMDVYEWDPSTDKYISVK 370

OryzasativaGBSSII_AAL58572.1_ PHYVKELASGPDKGVELDGILRTKPLETGIVNGMDVYEWNPATDQYISVK 370

ManihotesculentaGBSS_AET43458. PYYAQEVISGVERGVELDNFIR-KTGIAGIINGMDVQEWNPVTDKYIDIH 370

Eucalyptusgrandis_KCW72603.1_ PYYAEELVSGVAKGVELDNILR-RTGITGIVNGMDVQEWNPATDKYLDIH 375

PrunuspersicaGBSSII-1_AFA36447 PYYAQELISGEDKGVELDNIIR-KTGITGIINGMDVQEWNPARDKYLDVK 375

SolanumtuberosumGBSS_ABY89288. PYYAQELVSAVDKGVELDSVLR-KTCITGIVNGMDTQEWNPATDKYTDVK 369

ArabidopsisthalianaGBSS_AAM744 PYYAQELISGVDRGVELHKYLR-MKTVSGIINGMDVQEWNPSTDKYIDIK 372

PrunuspersicaGBSSII-2_AFA36448 PYYAEELVSTVEKGVELDNVIR-KAGILGIVNGMDVQEWNPLTDKYTAAK 380

Vitisvinifera_XP_002273608.1_ PYYAKELVSGVEKGVELDNILR-RTGITGIINGMDVQEWNPLTDKYISIK 376

PisumsativumGBSSIIb_CAC69955.1 PYYAQELVSGPDKGVELDNILR-RVGVTGIVNGMDVQEWNPSTDKYISIK 375

PisumsativumGBSSIIa_AAB26591.1 PHYAKELISGEDRGVELDNIIR-STGIIGIVNGMDNREWSPQTDRYIDVH 365

Chlamydomonasreinhardtii_AAC17 PNYATEIAADAAGGVELDTVIR-AKGIEGIVNGMDIEEWNPKTDKFLSVP 352

* *. *: : * **. :* **:**** :*.* *::

HordeumvulgareGBSSI_AHC55210.1 YDITTALEAKALNKEALQAEVGLPVDRKVPLVAFIGRLEEQKGPDVMIAA 414

OryzasativaGBSSI_AAF72562.1_ YDATTAIEAKALNKEALQAEAGLPVDRKIPLIAFIGRLEEQKGPDVMAAA 421

ZeamaysGBSSI_NP_001105001_ YDVSTAVEAKALNKEALQAEVGLPVDRNIPLVAFIGRLEEQKGPDVMAAA 415

HordeumvulgareGBSSII_BAJ99426. YNATTVAEARALNKEILQAEVGLPVDSSIPVIVFIGRLEEQKGSDILIAA 405

ZeamaysGBSSII_NP_001106039.1_ YDATTVTEARALNKERLQAEVGLPVDSSIPVIVFVGRLEEQKGSDILIAA 420

OryzasativaGBSSII_AAL58572.1_ YDATTVTEARALNKEMLQAEVGLPVDSSIPLIVFVGRLEEQKGSDILIAA 420

ManihotesculentaGBSS_AET43458. YDATTVMDAKPLLKEALQAEVGLPVDRNVPLIGFIGRLEEQKGSDIFVAA 420

Eucalyptusgrandis_KCW72603.1_ YDETTVMDAKPLLKEALQAAVGLPVDRNIPLIGFIGRLEEQKGSDILAAA 425

PrunuspersicaGBSSII-1_AFA36447 YDNTTVLDAKPLLKEALQAQVGLPVDRDIPVIGFIGRLEEQKGSDILVEA 425

SolanumtuberosumGBSS_ABY89288. YDITTVMDAKPLLKEALQAAVGLPVDKKVPLIGFIGRLEEQKGSDILVAA 419

ArabidopsisthalianaGBSS_AAM744 YDITTVTDAKPLIKEALQAAVGLPVDRDVPVIGFIGRLEEQKGSDILVEA 422

PrunuspersicaGBSSII-2_AFA36448 YDASTVTDAKPLLKEALQAEVGLPVDRDIPVIGFIGRLEEQKGSDILIEA 430

Vitisvinifera_XP_002273608.1_ YDASTVKEAKLLLKEALQAEAGLPVDRNIPVIGFIGRLEEQKGSDILVAA 426

PisumsativumGBSSIIb_CAC69955.1 YDASTVLEGKALLKEELQAEVGLPVDKNVPLIAFIGRLEEQKGSDILVEA 425

PisumsativumGBSSIIa_AAB26591.1 YNETTVTEAKPLLKGTLQAEIGLPVDSSIPLIGFIGRLEEQKGSDILVEA 415

Chlamydomonasreinhardtii_AAC17 YDQNSVYAGKAAAKEALQAELGLPVDPTAPLFAFIGRLEEQKGVDIILAA 402

*: .:. .: * *** ***** *:. *:******** *:: *

HordeumvulgareGBSSI_AHC55210.1 IPEILKE-EDVQIILLGTGKKKFEKLLKSMEEKFPGKVRAVVRFNAPLAH 463

OryzasativaGBSSI_AAF72562.1_ IPELMQ--EDVQIVLLGTGKKKFEKLLKSMEEKYPGKVRAVVKFNAPLAH 469

ZeamaysGBSSI_NP_001105001_ IPQLMEMVEDVQIVLLGTGKKKFERMLMSAEEKFPGKVRAVVKFNAALAH 465

HordeumvulgareGBSSII_BAJ99426. IPEFVE--ENVQIIVLGTGKKKMEEELMLLEVKYPQNARGIAKFNVPLAH 453

ZeamaysGBSSII_NP_001106039.1_ IPEFVG--ENVQIIVLGTGKKKMEEELTQLEVKYPNNARGIAKFNVPLAH 468

OryzasativaGBSSII_AAL58572.1_ IPEFVE--GNVQIIVLGTGKKKMEEELILLEVKYPNTARGLAKFNVPLAH 468

ManihotesculentaGBSS_AET43458. ISQLVE--HNVQIVILGTGKKKFEKQIEHLEVLYPDKARGVAKFNVPLAH 468

Eucalyptusgrandis_KCW72603.1_ IPKFIG--ENVQVVVLGTGKKTMERQLEELEIKYPSNARGVAKFNVPLAH 473

PrunuspersicaGBSSII-1_AFA36447 ISKFIG--EEVQIIILGTGKNYLETQIKQLEIKFPGKAVGVAKFNVPLAH 473

SolanumtuberosumGBSS_ABY89288. IHKFIG--LDVQIVVLGTGKKEFEQEIEQLEVLYPNKAKGVAKFNVPLAH 467

ArabidopsisthalianaGBSS_AAM744 ISKFMG--LNVQMVILGTGKKKMEAQILELEEKFPGKAVGVAKFNVPLAH 470

PrunuspersicaGBSSII-2_AFA36448 IPHFIK--ENVQIIVLGTGKKPMEKQLEQLEIKYPDKARGVAKFNVPLAH 478

Vitisvinifera_XP_002273608.1_ IPQFVT--ANVQIIVLGTGKKPMEKQLEQLEKKYPDNARGVAKFNVPLAH 474

PisumsativumGBSSIIb_CAC69955.1 IPQFIK--ENVQIVALGTGKKEMEKQLQQLEISYPDKARGVAKFNVPLAH 473

PisumsativumGBSSIIa_AAB26591.1 IAKFAD--ENVQIVVLGTGKKIMEKQIEVLEEKYPGKAIGITKFNSPLAH 463

Chlamydomonasreinhardtii_AAC17 LPKILAT-PKVQIAILGTGKAAYEKLVNAIGTKYKGRAKGVVKFSAPLAH 451

: .: .**: ***** * : : . .:.:*. .***

HordeumvulgareGBSSI_AHC55210.1 QMMAGADLLAVTSRFEPCGLIQLQGMRYGTPCVCASTGGLVDTIVEGKTG 513

OryzasativaGBSSI_AAF72562.1_ LIMAGADVLAVPSRFEPCGLIQLQGMRYGTPCACASTGGLVDTVIEGKTG 519

ZeamaysGBSSI_NP_001105001_ HIMAGADVLAVTSRFEPCGLIQLQGMRYGTPCACASTGGLVDTIIEGKTG 515

HordeumvulgareGBSSII_BAJ99426. MMFAGADFIIIPSRFEPCGLIQLQGMRYGVVPICSSTGGLVDTVREGVTG 503

ZeamaysGBSSII_NP_001106039.1_ MMFAGADFIIVPSRFEPCGLIQLQGMRYGVIPICSSTGGLVDTVEEGVTG 518

OryzasativaGBSSII_AAL58572.1_ MMFAGADFIIVPSRFEPCGLIQLQGMRYGVVPICSSTGGLVDTVKEGVTG 518

ManihotesculentaGBSS_AET43458. MITAGADFMLVPSRFEPCGLIQLHAMRYGTVPIVASTGGLVDTVKEGYTG 518

Eucalyptusgrandis_KCW72603.1_ MIIAGADFMLIPSRFEPCGLIQLQAMRYGTVPVVSSTGGLVDTIKEGFTG 523

PrunuspersicaGBSSII-1_AFA36447 MIIGGADFMLIPSRFEPCGLIQLHAMRYGTVPIVASTGGLVDTVKEGFTG 523

SolanumtuberosumGBSS_ABY89288. MITAGADFMLVPSRFEPCGLIQLHAMRYGTVPICASTGGLVDTVKEGYTG 517

ArabidopsisthalianaGBSS_AAM744 MITAGADFIIVPSRFEPCGLIQLHAMRYGTVPIVASTGGLVDTVKDGYTG 520

PrunuspersicaGBSSII-2_AFA36448 MITAGADFMLVPSRFEPCGLIQLHAMRYGTVPIVASTGGLVDTVKEGFTG 528

Vitisvinifera_XP_002273608.1_ MIIAGADFLLVPSRFEPCGLIQLHAMRYGTVPIVASTGGLVDTVKEGFTG 524

PisumsativumGBSSIIb_CAC69955.1 MMIAGADFILIPSRFEPCGLIQLQAMRYGTVPIVASTGGLVDTVKEGFTG 523

PisumsativumGBSSIIa_AAB26591.1 KIIAGADFIVIPSRFEPCGLVQLHAMPYGTVPIVSSTGGLVDTVKEGYTG 513

Chlamydomonasreinhardtii_AAC17 MLTAGADFMLVPSRFEPCGLIQLHAMHYGTVPVVASTGGLVDTVKEGVTG 501

: .***.: :.********:**:.* **. :********: :* **

HordeumvulgareGBSSI_AHC55210.1 FHMGRLSVDCNVVEPADVEKVATTLKRAVKVVGTPTYQEMVKNCMIQDLS 563

OryzasativaGBSSI_AAF72562.1_ FHMGRLSVDCKVVEPSDVKKVAATLKRAIKVVGTPAYEEMVRNCMNQDLS 569

ZeamaysGBSSI_NP_001105001_ FHMGRLSVDCNVVEPADVKKVATTLQRAIKVVGTPAYEEMVRNCMIQDLS 565

HordeumvulgareGBSSII_BAJ99426. FHMGSFNVEFETVDPADVTAVGSNVTRALKQYRTPVFHAMVQNCMAQDLS 553

ZeamaysGBSSII_NP_001106039.1_ FHMGSFNVECETVDPADVTAVASTVTRALMQYDTPAFHEMVQNCMAQDLS 568

OryzasativaGBSSII_AAL58572.1_ FHMGLFNVECETVDPVDVTAVASTVKRALKQYNTPAFQEMVQNCMAQDLS 568

ManihotesculentaGBSS_AET43458. FQMGALRVECDKIDSADVAAIVKTVARALGTYATAALREMILNCMAQDLS 568

Eucalyptusgrandis_KCW72603.1_ FQMGGFSLDCDVIDPADVDAVARTVKRALATYGTAAMKEMISNCMAQDLS 573

PrunuspersicaGBSSII-1_AFA36447 FQMGAFNVVCDEVDPADVTAIATTVKRALAAYGTPALNEMIQNCMAQDLS 573

SolanumtuberosumGBSS_ABY89288. FHMGAFNVECDVVDPADVLKIVTTVARALAVYGTLAFAEMIKNCMSEELS 567

ArabidopsisthalianaGBSS_AAM744 FHIGRFNVKCEVVDPDDVIATAKAVTRAVAVYGTSAMQEMVKNCMDQDFS 570

PrunuspersicaGBSSII-2_AFA36448 FQMGGFNVECEVVDPADVQAIATTVTRALGTYGTPAFTEIIGNCMAQDLS 578

Vitisvinifera_XP_002273608.1_ FQMGSFSVECDAVDPADATAVATSVKRALATYGTPALTEMVQNCMAQDLS 574

PisumsativumGBSSIIb_CAC69955.1 FHMGSFNVKCDAVDPVDVDAIPKTVTKALGVYGTSAFAEMIKNCMAQELS 573

PisumsativumGBSSIIa_AAB26591.1 FHAGPFDVECEDVDPDDVDKLAATVKRALKTYGTQAMKQIILNCMAQNFS 563

Chlamydomonasreinhardtii_AAC17 FHMGALNP--DKLDEADADALAATVRRASEVFAGGRYPEMVANCISQDLS 549

*: * : . :: *. : :* :: **: :::*

HordeumvulgareGBSSI_AHC55210.1 WKGPAKNWEDVLLELGVEGSEPGIV-GEEIAPLAMENVAAP--------- 603

OryzasativaGBSSI_AAF72562.1_ WKGPAKNWENVLLGLGVAGSAPGIE-GDEIAPLAKENVAAP--------- 609

ZeamaysGBSSI_NP_001105001_ WKGPAKNWENVLLSLGVAGGEPGVE-GEEIAPLAKENVAAP--------- 605

HordeumvulgareGBSSII_BAJ99426. WKGPAKKWEEALLSLGVEGSQPGIE-GEEIAPLAKQNVATP--------- 593

ZeamaysGBSSII_NP_001106039.1_ WKGPAKEWEEVLLGLGVEGSRAGIDDAEEIAPLAKENVATP--------- 609

OryzasativaGBSSII_AAL58572.1_ WKGPAKKWEEVLLGLGVEGSQPGIE-GEEVAPLAKENVATP--------- 608

ManihotesculentaGBSS_AET43458. WKGPARMWEKMLLDLEVTGSEPGTE-GEEIAPLAKENVPTP--------- 608

Eucalyptusgrandis_KCW72603.1_ WKGPAGLWEKMLLGLDIAGGEPGVE-GEEVAPLAKENIATP--------- 613

PrunuspersicaGBSSII-1_AFA36447 WKGPSKQWEKMLLSLEVAGGEPGIE-GEEIAPLAKENVPTP--------- 613

SolanumtuberosumGBSS_ABY89288. WKAPAKKWETLLLGLGASGSEPGVE-GEEIAPLAKENVATP--------- 607

ArabidopsisthalianaGBSS_AAM744 WKGPARLWEKVLLSLNVAGSEAGTE-GEEIAPLAKENVATP--------- 610

PrunuspersicaGBSSII-2_AFA36448 WKGPAKKWEEVLLNLGVVDSEPGID-GEEIAPLAKENIATP--------- 618

Vitisvinifera_XP_002273608.1_ WKGPAKKWEEVLLSLGVPGSEPGID-GEEIAPLSKENVATP--------- 614

PisumsativumGBSSIIb_CAC69955.1 WKGPAKKWEEVLLNLGVPDSEPGID-GQEIAPQAKENVATP--------- 613

PisumsativumGBSSIIa_AAB26591.1 WKKPAKLWEKALLNLEVTGNVAGID-GDEIAPLAKENVATP--------- 603

Chlamydomonasreinhardtii_AAC17 WSKPAQKWEGLLEEVVYGKGGVATAKKEEIKVPVAEKIPGDLPAVSYAPN 599

*. *: ** * : . . :*: :::.

HordeumvulgareGBSSI_AHC55210.1 --------------------------------------------------

OryzasativaGBSSI_AAF72562.1_ --------------------------------------------------

ZeamaysGBSSI_NP_001105001_ --------------------------------------------------

HordeumvulgareGBSSII_BAJ99426. --------------------------------------------------

ZeamaysGBSSII_NP_001106039.1_ --------------------------------------------------

OryzasativaGBSSII_AAL58572.1_ --------------------------------------------------

ManihotesculentaGBSS_AET43458. --------------------------------------------------

Eucalyptusgrandis_KCW72603.1_ --------------------------------------------------

PrunuspersicaGBSSII-1_AFA36447 --------------------------------------------------

SolanumtuberosumGBSS_ABY89288. --------------------------------------------------

ArabidopsisthalianaGBSS_AAM744 --------------------------------------------------

PrunuspersicaGBSSII-2_AFA36448 --------------------------------------------------

Vitisvinifera_XP_002273608.1_ --------------------------------------------------

PisumsativumGBSSIIb_CAC69955.1 --------------------------------------------------

PisumsativumGBSSIIa_AAB26591.1 --------------------------------------------------

Chlamydomonasreinhardtii_AAC17 TLKPVSASVEGNGAAAPKVGTTAPAMGAWRATTPSGPSPAAATPKVTTYK 649

HordeumvulgareGBSSI_AHC55210.1 --------------------------------------------------

OryzasativaGBSSI_AAF72562.1_ --------------------------------------------------

ZeamaysGBSSI_NP_001105001_ --------------------------------------------------

HordeumvulgareGBSSII_BAJ99426. --------------------------------------------------

ZeamaysGBSSII_NP_001106039.1_ --------------------------------------------------

OryzasativaGBSSII_AAL58572.1_ --------------------------------------------------

ManihotesculentaGBSS_AET43458. --------------------------------------------------

Eucalyptusgrandis_KCW72603.1_ --------------------------------------------------

PrunuspersicaGBSSII-1_AFA36447 --------------------------------------------------

SolanumtuberosumGBSS_ABY89288. --------------------------------------------------

ArabidopsisthalianaGBSS_AAM744 --------------------------------------------------

PrunuspersicaGBSSII-2_AFA36448 --------------------------------------------------

Vitisvinifera_XP_002273608.1_ --------------------------------------------------

PisumsativumGBSSIIb_CAC69955.1 --------------------------------------------------

PisumsativumGBSSIIa_AAB26591.1 --------------------------------------------------

Chlamydomonasreinhardtii_AAC17 PALPATAKPKTAGLKLAGEASTTSTSENGAASNGNGNGASASKTSAAKPL 699

HordeumvulgareGBSSI_AHC55210.1 ---------

OryzasativaGBSSI_AAF72562.1_ ---------

ZeamaysGBSSI_NP_001105001_ ---------

HordeumvulgareGBSSII_BAJ99426. ---------

ZeamaysGBSSII_NP_001106039.1_ ---------

OryzasativaGBSSII_AAL58572.1_ ---------

ManihotesculentaGBSS_AET43458. ---------

Eucalyptusgrandis_KCW72603.1_ ---------

PrunuspersicaGBSSII-1_AFA36447 ---------

SolanumtuberosumGBSS_ABY89288. ---------

ArabidopsisthalianaGBSS_AAM744 ---------

PrunuspersicaGBSSII-2_AFA36448 ---------

Vitisvinifera_XP_002273608.1_ ---------

PisumsativumGBSSIIb_CAC69955.1 ---------

PisumsativumGBSSIIa_AAB26591.1 ---------

Chlamydomonasreinhardtii_AAC17 VSAATRKSA 708

Full sequence alignment used to produce Fig S4B
(region shown in figure highlighted yellow)

AtSS1 MASLQISGSVKFEPFVGFNRIRHFRPIASLGFPRFRRRFSIGRSLLLRRSSSFSGDSRES 60

HvSSI MAATGVG-AGCLAPSVRLRADPAARATACVVRARLRR---------VARGRYVAELSREG 50

AtGBSS MATVTASSNFVSRT--SLFNNHGASSCSDVAQITLKG----------------------- 35

OsGBSS1 MSALTTSQLATSATGFGIADRSAPSSLLRHGFQGLKP----------------------- 37

*:: . . : . ::

AtSS1 DEERFITDAERDGSGSVLGFQLTPPGDQQTVSTSTGEITHHEEKKEAIDQIVMADFGVPG 120

HvSSI PAAR---PAQQLAPPVVPGFLAPPPPAPAQSPAPTQPPLPDAGVGELAPDLLLEGIAEDS 107

AtGBSS ---------QSLTHCGLRSFNMVDNLQRRSQAKPVSAKSSKRSSK-VKTAGKIVCEKG-- 83

OsGBSS1 ---------RSPAGGDATSLSVTT----SARATPKQQRSVQRGSRRFPSVVVYATGAG-- 82

. .: . . .

AtSS1 NRAVEEGAAEVG--IPSGK----AEVVNNLVFVTSEAAPYSKTGGLGDVCGSLPIALAGR 174

HvSSI IDTIVVAASEQDSEIMDANDQPLAKVTRSIVFVTGEAAPYAKSGGLGDVCGSLPIALAAR 167

AtGBSS ---------------------------MSVIFIGAEVGPWSKTGGLGDVLGGLPPALAAR 116

OsGBSS1 ---------------------------MNVVFVGAEMAPWSKTGGLGDVLGGLPPAMAAN 115

.::*: .* .*::*:****** *.** *:*..

AtSS1 GHRVMVISPRYLNGTAADKNYARAKDLGIRVTVNCFGGSQEVSFYHEYRDGVDWVFVDHK 234

HvSSI GHRVMVVMPRYLNGTS-DKNYAKALYTGKHIKIPCFGGSHEVTFFHEYRDNVDWVFVDHP 226

AtGBSS GHRVMTICPRY-------DQYKDAWDTCVVVQIKVGDKVENVRFFHCYKRGVDRVFVDHP 169

OsGBSS1 GHRVMVISPRY-------DQYKDAWDTSVVAEIKVADRYERVRFFHCYKRGVDRVFIDHP 168

*****.: *** .:* * : . ..* *:* *: .** **:**

AtSS1 SYHRPGN------PYGDSK-GAFGDNQFRFTLLCHAACEAPLVLPLGG-----FTYGEKS 282

HvSSI SYHRPGS------LYGDNF-GAFGDNQFRYTLLCYAACEAPLILELGG-----YIYGQSC 274

AtGBSS IFLAKVVGKTGSKIYGPITGVDYNDNQLRFSLLCQAALEAPQVLNLNSSKYFSGPYGEDV 229

OsGBSS1 SFLEKVWGKTGEKIYGPDTGVDYKDNQMRFSLLCQAALEAPRILNLNNNPYFKGTYGEDV 228

: ** : ***:*::*** ** *** :* *.. **:.

AtSS1 LFLVNDWHAGLVPILLAAKYRPYGVYKDARSILIIHNLAHQGVEPAATYTNLGLPSEWYG 342

HvSSI MFVVNDWHASLVPVLLAAKYRPYGVYRDSRSTLVIHNLAHQGVEPASTYPDLGLPPEWYG 334

AtGBSS VFVANDWHTALLPCYLKSMYQSRGVYMNAKVVFCIHNIAYQGRFAFDDYSLLNLPISFKS 289

OsGBSS1 VFVCNDWHTGPLASYLKNNYQPNGIYRNAKVAFCIHNISYQGRFAFEDYPELNLSERFRS 288

:*: ****:. :. * *:. *:* ::: : ***:::** . *. *.*. : .

AtSS1 AVGWVFPTWARTHALDTGEAVNVLKGAIVTSDRIITVSQGYAWEITTVEGGYGLQDLLSS 402

HvSSI ALEWVFPEWARRHALDKGEAVNFLKGAVVTADRIVTVSQGYSWEVTTAEGGQGLNELLSS 394

AtGBSS SFDFMD----GYEKPVKGRKINWMKAAILEAHRVLTVSPYYAQELISG-VDRGVELHKYL 344

OsGBSS1 SFDFID----GYDTPVEGRKINWMKAGILEADRVLTVSPYYAEELISG-IARGCELDNIM 343

:. :: . *. :* :*..:: :.*::*** *: *: : * :

AtSS1 RKSVINGITNGINVDEWNPSTDEHIPFHYSADDVS-EKIKCKMALQKELGLPIRPECPMI 461

HvSSI RKSVLNGIVNGIDINDWNPTTDKCLPHHYSVDDLS-GKAKCKAELQRELGLPVREDVPLI 453

AtGBSS RMKTVSGIINGMDVQEWNPSTDKYIDIKYDITTVTDAKPLIKEALQAAVGLPVDRDVPVI 404

OsGBSS1 RLTGITGIVNGMDVSEWDPSKDKYITAKYDATTAIEAKALNKEALQAEAGLPVDRKIPLI 403

* . :.** **:::.:*:*:.*: : :*. * * ** ***: . *:*

AtSS1 GFIGRLDYQKGIDLIQTAGPDLMVDDIQFVMLGSGDPKYESWMRSMEETYRDKFRGWVGF 521

HvSSI GFIGRLDYQKGIDLIKMAIPDLMREDVQFVMLGSGDPVFEGWMRSTESSYKDKFRGWVGF 513

AtGBSS GFIGRLEEQKGSDILVEAISKFMGLNVQMVILGTGKKKMEAQILELEEKFPGKAVGVAKF 464

OsGBSS1 AFIGRLEEQKGPDVMAAAIPELMQEDVQIVLLGTGKKKFEKLLKSMEEKYPGKVRAVVKF 463

.*****: *** *:: * ..:* ::*:*:**:*. * : . *..: .* . . *

AtSS1 NVPISHRITAGCDILLMPSRFEPCGLNQLYAMRYGTIPVVHGTGGLRDTVENFNPYAEGG 581

HvSSI SVPVSHRITAGCDILLMPSRFEPCGLNQLYAMQYGTVPVVHGTGGLRDTVETFNPFGAKG 573

AtGBSS NVPLAHMITAGADFIIVPSRFEPCGLIQLHAMRYGTVPIVASTGGLVDTVKDGYTGFHIG 524

OsGBSS1 NAPLAHLIMAGADVLAVPSRFEPCGLIQLQGMRYGTPCACASTGGLVDTVIEGKTGFHMG 523

..*::* * **.*.: :********* ** .*:*** .**** *** . *

AtSS1 AGTGTGWVFTPLSKDSMVSALRLAAATYREYKQSWEGLMRRGMTRNYSWENAAVQYEQVF 641

HvSSI E-EGTGWAFSPLTVEKMLWALRTAISTFREHKPSWEGLMKRGMTKDHTWDHAAEQYEQIF 632

AtGBSS RFNVKCEVVDPDDVIATAKAVTRAVAVYG--TSAMQEMVKNCMDQDFSWKGPARLWEKVL 582

OsGBSS1 RLSVDCKVVEPSDVKKVAATLKRAIKVVG--TPAYEEMVRNCMNQDLSWKGPAKNWENVL 581

.. * :: * . . : : :::. * :: :*. .* :*:::

AtSS1 QWVFMDPPYVS----------------- 652

HvSSI EWAFVDQPYVM----------------- 643

AtGBSS LSLNVAGSEAGTEGEEIAPLAKENVATP 610

OsGBSS1 LGLGVAGSAPGIEGDEIAPLAKENVAAP 609

: .
