# Supplementary figures and images for: PROTEIN TARGETING TO STARCH Is Required for Localising GRANULE-BOUND STARCH SYNTHASE to Starch Granules and for Normal Amylose Synthesis in Arabidopsis
Source: PLoS Biol. 2015 Feb 24;13(2):e1002080. doi: 10.1371/journal.pbio.1002080 (PMC4339375; doi:10.1371/journal.pbio.1002080)

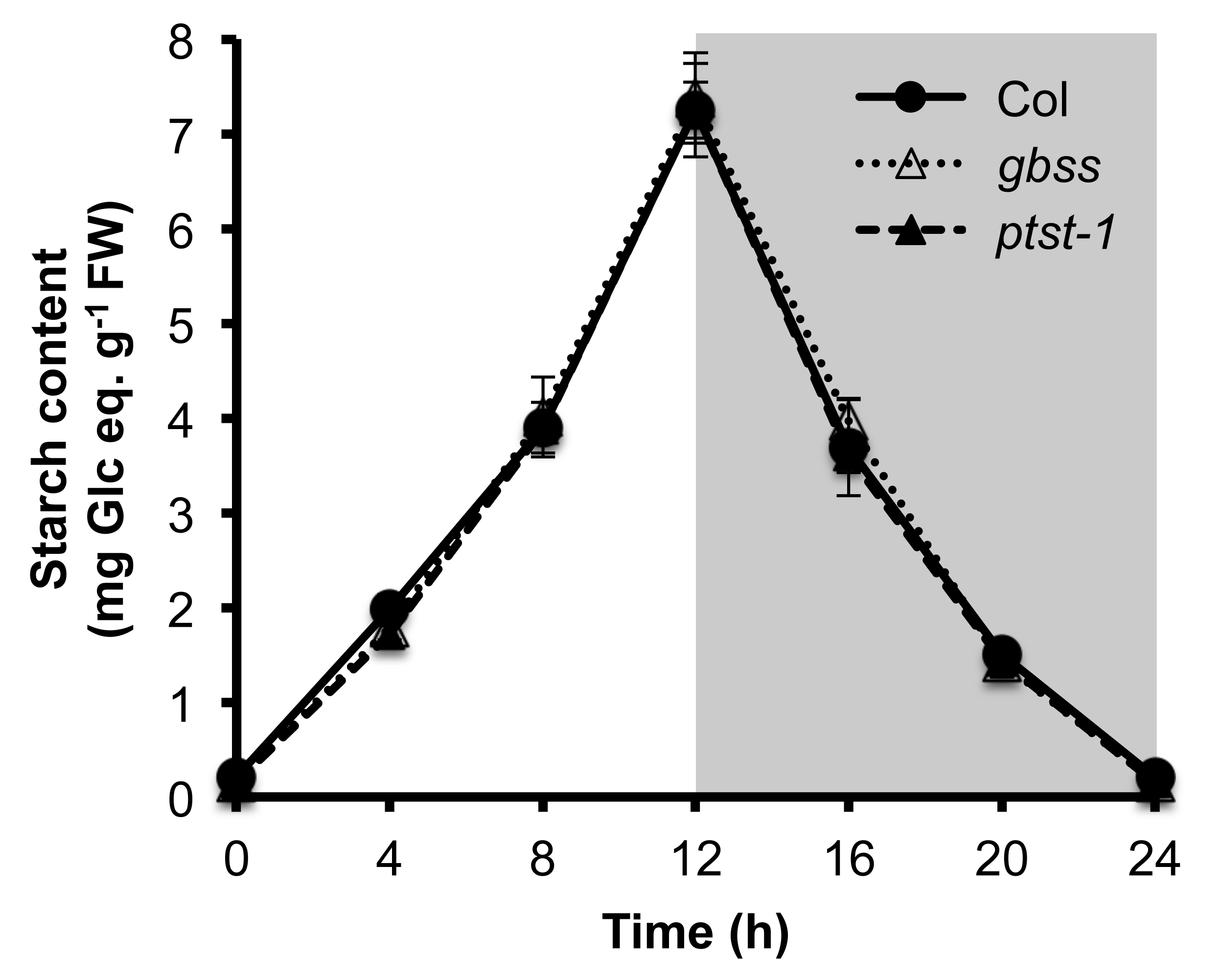

Supplement: S1 Fig — Plants were grown under a 12-h light (0–12 h) and 12-h dark (12–24 h) regime. Entire Arabidopsis rosettes were harvested every 4 h and starch was quantified. Values are the mean ± standard error of the mean (SEM) from 4–5 plants. Note that values at the 0-h time point are replotted from the 24-h time point. No significant differences between mutants and wild type (Col) at p < 0.05 were observed at any time point. Numerical data used to generate the plot are provided in S5 Data. (TIF) [file pbio.1002080.s007.tif]

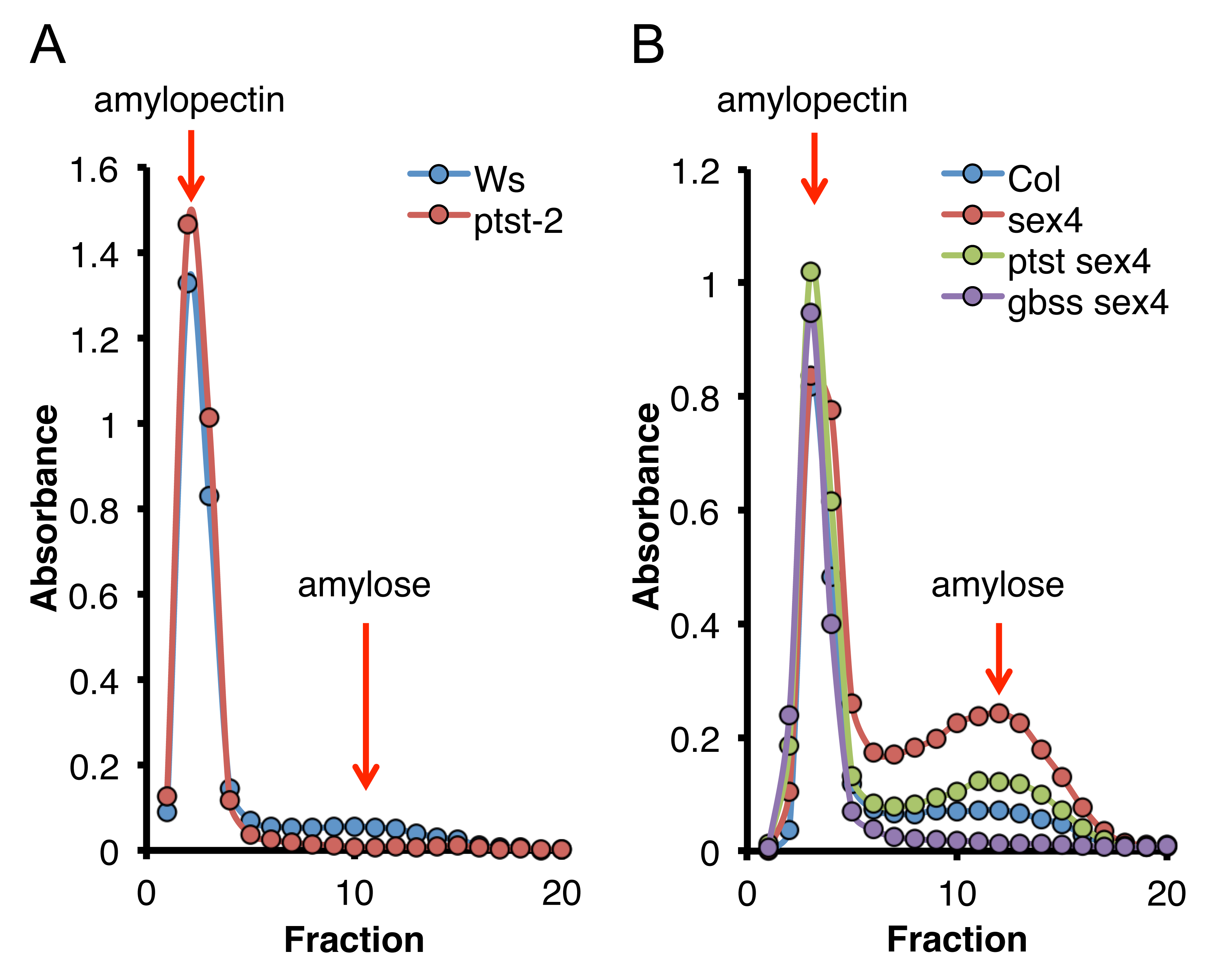

Supplement: S2 Fig — Purified granules were dissolved in 0.5 M NaOH and separated over a Sepharose CL-2B column. Fractions were mixed with an iodine solution, and absorbance was recorded at 595 nm. (A) Wild-type granules (Ws) versus ptst-2 starch granules. (B) Wild-type granules (Col) versus sex4, ptst sex4, and gbss sex4 starch granules. Numerical data used to generate the plots are provided in S5 Data. (TIF) [file pbio.1002080.s008.tif]

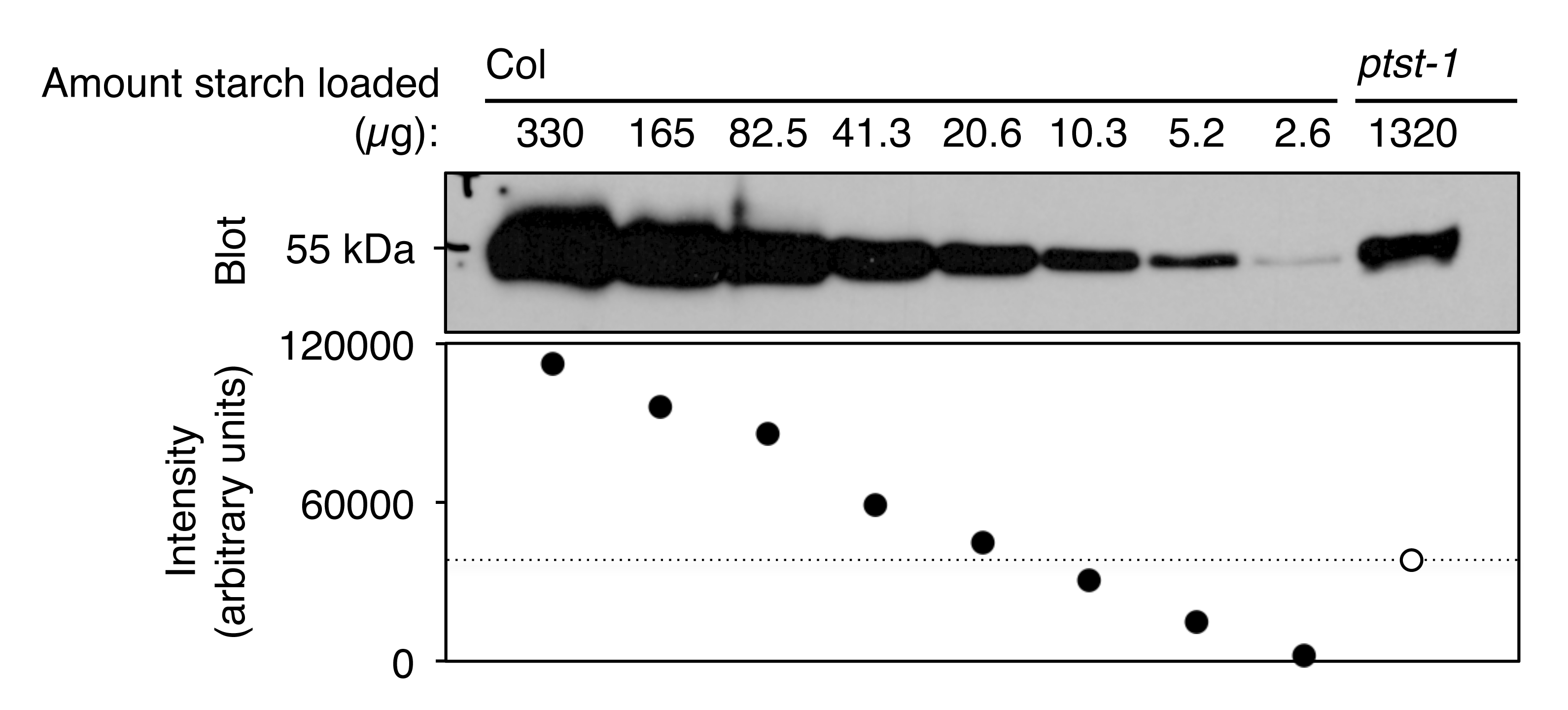

Supplement: S3 Fig — Granule-bound proteins extracted from purified Arabidopsis ptst starch granules, and a dilution series of proteins from wild-type starch, were separated by SDS-PAGE. GBSS was detected by immunoblotting using a GBSS-specific antiserum. The equivalent mass of starch loaded is indicated above each lane, and the corresponding band intensity calculated using ImageJ densitometry software is indicated below. Numerical data used to generate the plot (band intensity) are provided in S5 Data. (TIF) [file pbio.1002080.s009.tif]

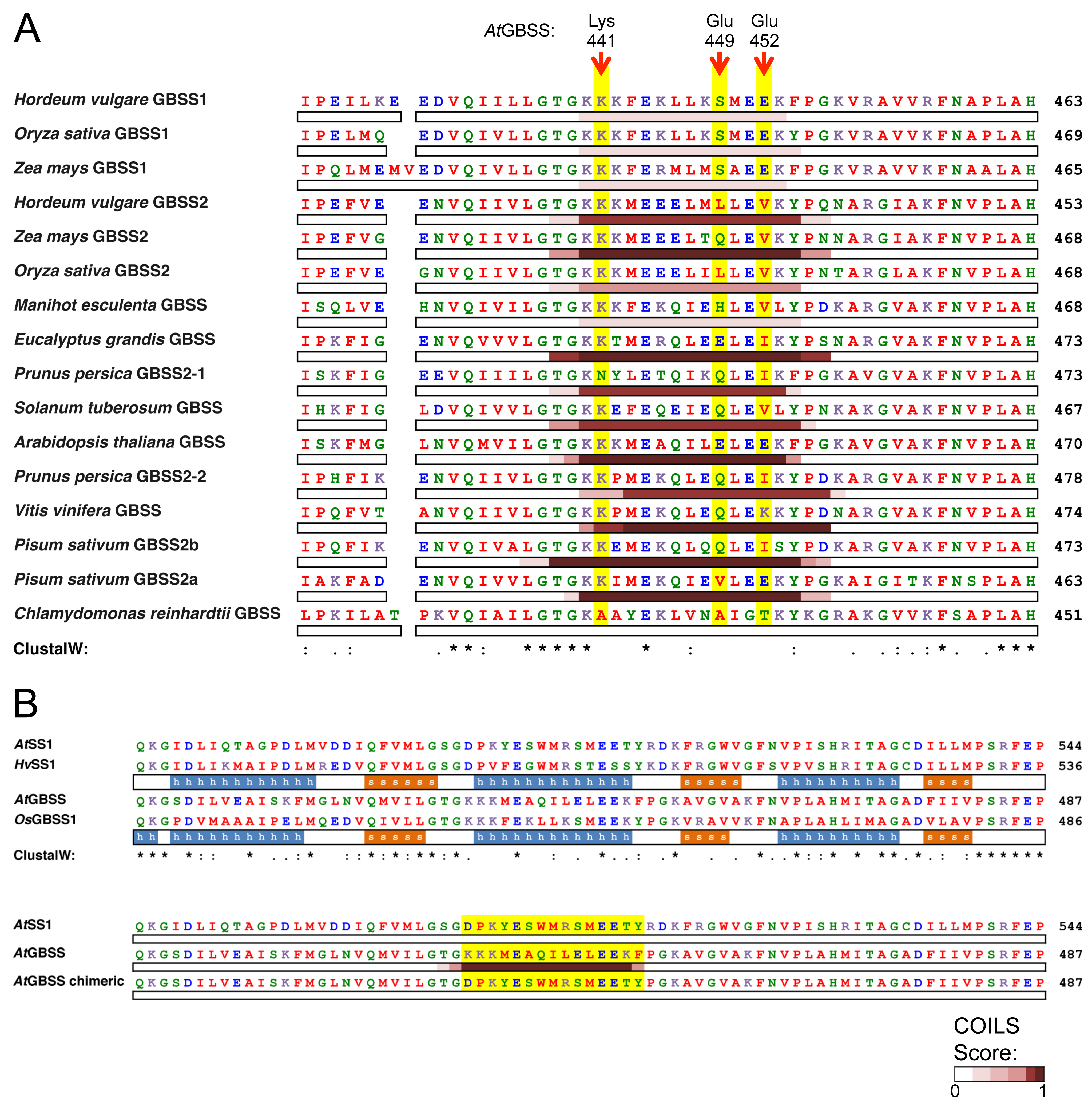

Supplement: S4 Fig — All alignments were generated using ClustalW, and the region surrounding the predicted coiled coil of GBSS is shown. (A). Sequence alignment of GBSS sequences from representative species of Viridiplantae. The COILS prediction scores for each individual sequence are indicated below the sequence. Amino acid positions containing Lys441, Glu449, and Glu452 are highlighted in yellow. (B) Generation of the chimeric protein between GBSS and SS1, which does not have a predicted coiled coil. The top panel shows the sequence alignment for A. thaliana GBSS and SS1 proteins, together with barley SS1 (HvSS1) and rice GBSS1 (OsGBSS1) proteins. Structural features from the solved HvSS1 and OsGBSS1 structures are depicted below the respective sequences, where helices (h) are represented in blue and sheets (s) are represented in orange. The bottom panel shows the sequence of the chimeric GBSS protein, where the helix containing the coiled coil from AtGBSS has been swapped with the homologous helix on AtSS1, together with an indication of COILS prediction scores. The swapped regions are highlighted in yellow. Full alignments used to generate these figures are provided in S6 Data. (TIF) [file pbio.1002080.s010.tif]

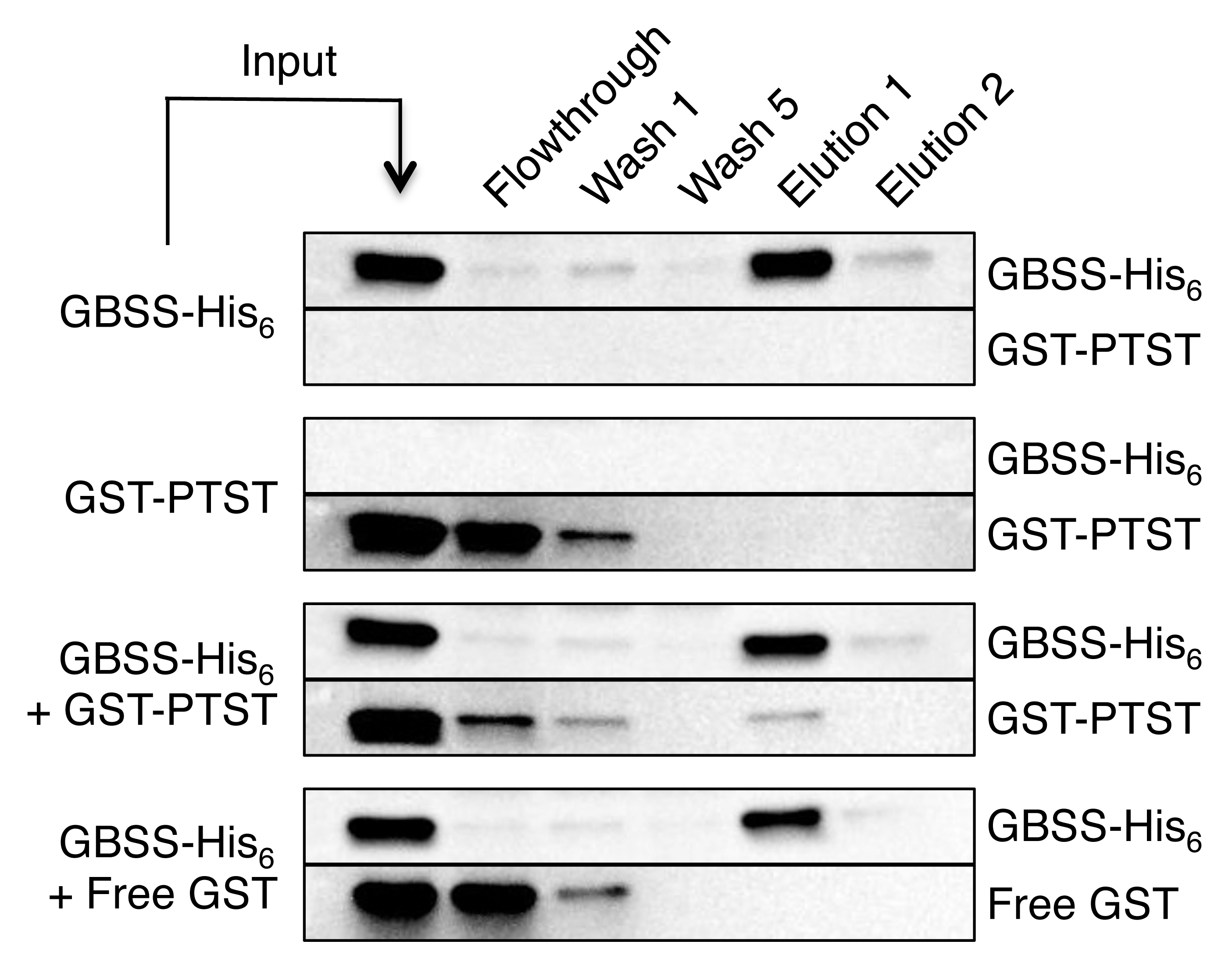

Supplement: S5 Fig — Purified recombinant proteins (1 μg each; Input) were incubated together with a Ni2+-NTA resin. Unbound proteins were removed in the supernatant (Flowthrough), and the resin was washed five times. Bound proteins were eluted twice with high imidazole. Proteins were detected by immunoblotting with anti-His for GBSS-His6, and anti-GST for GST-PTST, and free GST. (TIF) [file pbio.1002080.s011.tif]

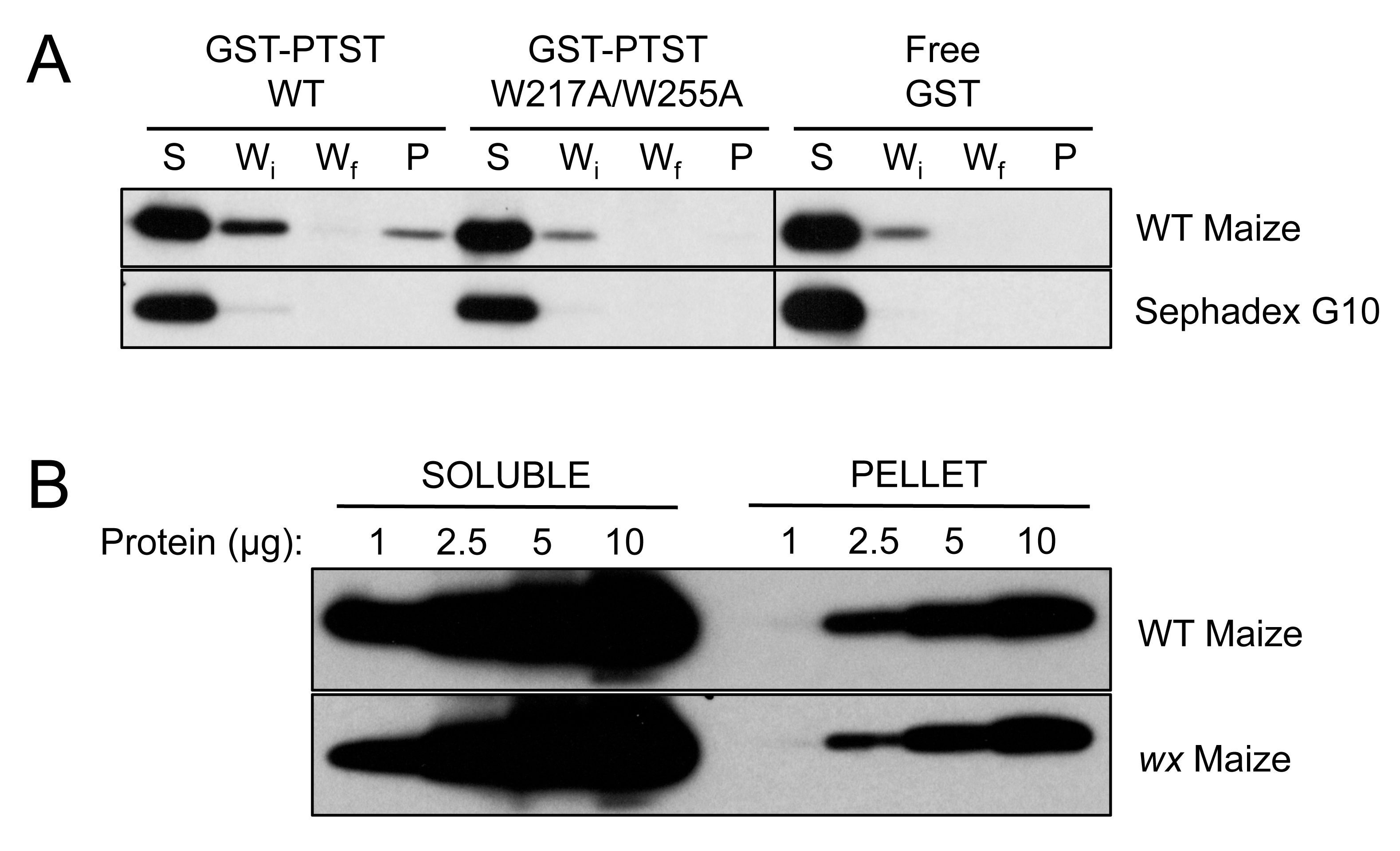

Supplement: S6 Fig — (A) Purified GST-PTST, the W217A/W255A variant, and free GST (1 μg each) was incubated with wild-type (WT) maize starch, or Sephadex G-10 as a non-glucan control. After centrifugation, unbound proteins were collected in the soluble fraction. After three washes, bound proteins were eluted from the starch pellet. Protein was detected in the soluble (S) and pellet (P) fractions, as well as the initial and final washes (Wi and Wf) by immunoblotting with anti-GST. (B) The indicated amounts of recombinant GST-PTST protein were incubated with wild-type (WT) or waxy (wx) maize starch. Starch binding assay was carried out as described for (A). Unbound proteins were detected in the supernatant (Soluble), while bound proteins were detected in the pellet fraction (Pellet). The amount of bound proteins increased as the amount of protein used in the assay increased. Starch was therefore not limiting for the amounts of protein tested. (TIF) [file pbio.1002080.s012.tif]

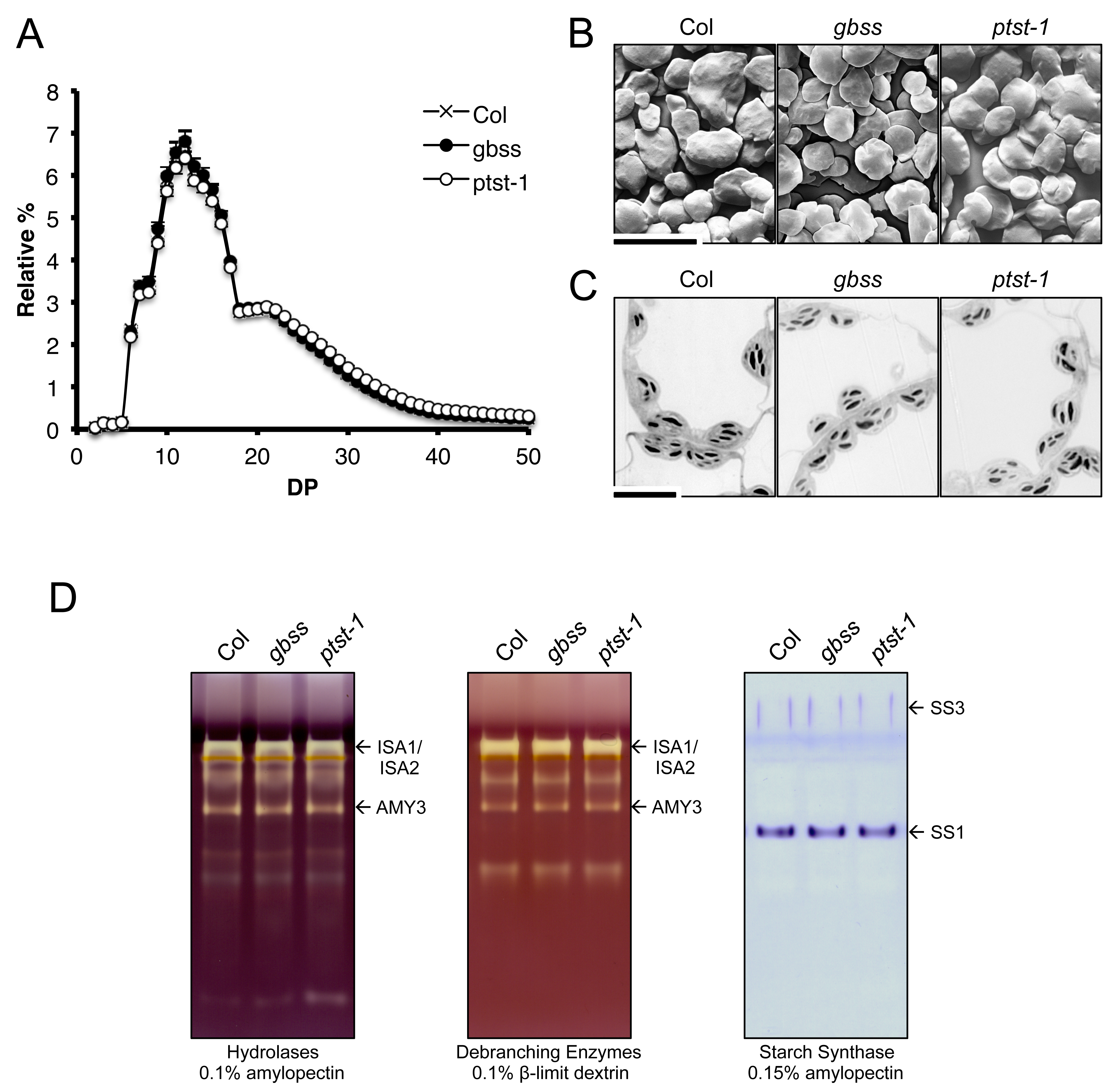

Supplement: S7 Fig — (A) Amylopectin chain length distribution is not affected by the loss of PTST. Starch was debranched enzymatically and prior to analysis with high performance anion exchange chromatography (HPAEC-PAD). The area of individual peaks corresponding to chains with a given degree of polymerisation (DP) was expressed as a percentage relative to the summed peak area for DP 2–50. Values represent mean ± standard error of four biological replicates. No significant differences were detected at p < 0.05 between wild-type and ptst mutant at any DP. Numerical data used to generate the plot are provided in S5 Data. (B) ptst mutants produce starch with normal granule morphology. Starch granules were purified from Arabidopsis rosettes and examined under a scanning electron microscope (SEM). Bar = 5 μm. (C) The number of starch granules per chloroplast is not affected by the loss of PTST. Resin-embedded leaf tissue was sectioned and stained with toluidine blue prior to light microscopy. Bar = 10 μm. (D) Zymogram analysis of soluble enzymatic activities in the ptst and gbss mutants. Soluble protein extracts of Arabidopsis leaves were separated on native PAGE gels containing 0.1% (w/v) amylopectin for visualising hydrolytic activities, 0.1% (w/v) β-limit dextrin for visualising debranching enzyme activities, and 0.15% (w/v) amylopectin for starch synthase activities. Equal amounts of protein were loaded per lane (30 μg). Following electrophoresis, gels were incubated in activity medium, and bands were observed by staining with iodine solution. The starch synthase activity gel was extensively destained in water. No differences were observed in the banding pattern between mutants and wild type (Col). (TIF) [file pbio.1002080.s013.tif]

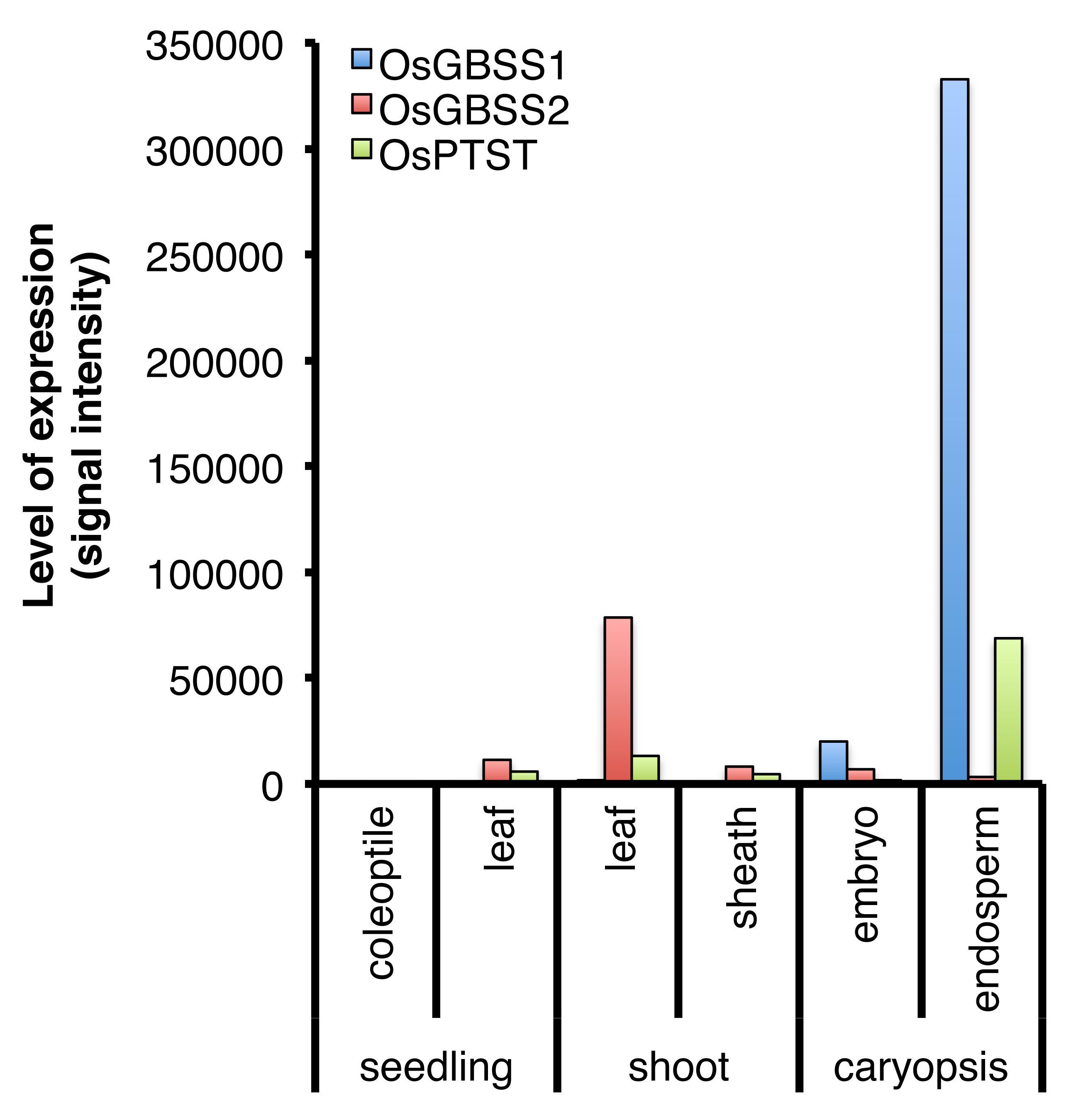

Supplement: S8 Fig — Data was obtained from the Genevestigator database, consisting of curated Rice Genome 51K array experiments. As expected, GBSS1 was mainly expressed in the endosperm, while GBSS2 was mainly expressed in leaf tissue. PTST was expressed in both leaf and endosperm tissues. Numerical data used to generate the plot are provided in S5 Data. (TIF) [file pbio.1002080.s014.tif]
