# Supplementary material for: PROTEIN TARGETING TO STARCH Is Required for Localising GRANULE-BOUND STARCH SYNTHASE to Starch Granules and for Normal Amylose Synthesis in Arabidopsis
Source: PLoS Biol. 2015 Feb 24;13(2):e1002080. doi: 10.1371/journal.pbio.1002080 (PMC4339375; doi:10.1371/journal.pbio.1002080)
Supplement: S1 Table — (DOCX) [file pbio.1002080.s015.docx]

| **TABLE S1**  **T-DNA insertion lines used in this study** | | | |  |
| --- | --- | --- | --- | --- |
| Genotype | Ecotype | Insertion location | Insertion sequence | |
| **Lines characterised by this study** | | | | |
| *ptst-1* (SALK_025022) | Col | Exon 3 | TGTCTAAGCGTCAA// (5 bp) aagatttactagat | |
| *ptst-2* (FLAG_622E03) | Ws | Exon 9 | gaaatttaggtacg (0 bp) // AGAAACCCCAACCC | |
| Genotype | Ecotype | Reference |  | |
| **Previously characterised lines** | | | | |
| *dpe1*  (GABI_339B11) | Col | [50] |  | |
| *gbss*  (GABI_914G01) | Col | [9] |  | |
| *sex4*  (SALK_102567) | Col | [32] |  | |
| For the lines characterised by this study, the location of the T-DNA insertion is shown together with the sequence flanking the insertion. The portion of the sequence corresponding to the *PTST* gene is displayed in lower case and the T-DNA-derived sequence in upper case. The length of the intervening sequence (not derived from either the T-DNA or the gene) is shown in parenthesis. | | | |  |
